# Genomic analysis reveals neutral and adaptive patterns that challenge the current management regime for East Atlantic cod *Gadus morhua L*

**DOI:** 10.1111/eva.13070

**Published:** 2020-09-05

**Authors:** Torild Johansen, François Besnier, María Quintela, Per Erik Jorde, Kevin A. Glover, Jon‐Ivar Westgaard, Geir Dahle, Sigbjørn Lien, Matthew P. Kent

**Affiliations:** ^1^ Institute of Marine Research (IMR) Tromsø Norway; ^2^ Institute of Marine Research (IMR) Bergen Norway; ^3^ Institute of Marine Research (IMR) His Norway; ^4^ Department of Biology University of Bergen Bergen Norway; ^5^ Department of Animal and Aquaculture Sciences Faculty of Biosciences Centre for Integrative Genetics Norwegian University of Life Sciences Ås Norway

**Keywords:** chromosomes, haplotype maps, inversions, managements, White Sea cod

## Abstract

Challenging long‐held perceptions of fish management units can help to protect vulnerable stocks. When a fishery consisting of multiple genetic stocks is managed as a single unit, overexploitation and depletion of minor genetic units can occur. Atlantic cod (*Gadus morhua*) is an economically and ecologically important marine species across the North Atlantic. The application of new genomic resources, including SNP arrays, allows us to detect and explore novel structure within specific cod management units. In Norwegian waters, coastal cod (i.e. those not undertaking extensive migrations) are divided into two arbitrary management units defined by ICES: one between 62° and 70°N (Norwegian coastal cod; NCC) and one between 58° and 62°N (Norwegian coastal south; NCS). Together, these capture a fishery area of >25,000 km^2^ containing many spawning grounds. To assess whether these geographic units correctly represent genetic stocks, we analysed spawning cod of NCC and NCS for more than 8,000 SNPs along with samples of Russian White Sea cod, north‐east Arctic cod (NEAC: the largest Atlantic stock), and outgroup samples representing the Irish and Faroe Sea's. Our analyses revealed large differences in spatial patterns of genetic differentiation across the genome and revealed a complex biological structure within NCC and NCS. Haplotype maps from four chromosome sets show regional specific SNP indicating a complex genetic structure. The current management plan dividing the coastal cod into only two management units does not accurately reflect the genetic units and needs to be revised. Coastal cod in Norway, while highly heterogenous, is also genetically distinct from neighbouring stocks in the north (NEAC), west (Faroe Island) and the south. The White Sea cod are highly divergent from other cod, possibly yielding support to the earlier notion of subspecies rank.

## 1 INTRODUCTION

In marine fisheries, stocks or management units are often defined by national borders or economic zones that do not always reflect the true biological or genetic units (Kerr et al., 2016; Reiss, Hoarau, Dickey‐Collas, & Wolff, 2009; Saha et al., 2015). Depletion and overexploitation of vulnerable stocks can occur where a fishery consisting of multiple genetic units or stocks is managed as a single unit (Allendorf, England, Luikart, Ritchie, & Ryman, 2008; Hauser, Adcock, Smith, Ramírez, & Carvalho, 2002; Ruzzante, Taggart, & Cook, 1999). Eroding a vulnerable stock may reduce its overall genetic variation and, in turn, impair the overexploited stocks potential to adapt to environmental change (Hauser et al., 2002). New genomic tools allow for a more accurate assessment of population genetic structure and have been successfully applied to redefine independent stocks and management units within fisheries (Hauser, Waples, & Carvalho, 2008; Reiss et al., 2009).

Atlantic cod (*Gadus morhu*a L) is an economically important demersal fish distributed across most of the North Atlantic Ocean (Brander, 1995) where it occurs in both offshore and in coastal areas. Cod from the offshore and coastal areas are typically managed as a single stock, or the coastal cod are simply a subunit relative to the offshore stock component (Berg & Albert, 2003; Bradbury et al., 2013; ICES, 2019; Johansen et al., 2017; Kerr, Cadrin, & Kovach, 2014). However, genetic differences have been detected between offshore and coastal cod in several regions, including the Gulf of Maine (Kerr et al., 2014; Kovach, Breton, Berlinsky, Maceda, & Wirgin, 2010), Eastern Canada (Bradbury et al., 2013; Ruzzante et al., 1999), Iceland (Berg et al., 2016; Pampoulie et al., 2012), Greenland (Pampoulie et al., 2011), the Faroe Islands (Nielsen, Hemmer‐Hansen, et al., 2009) and Norway (Wennevik, Jørstad, Dahle, & Fevolden, 2008). Spies et al. (2018) and Kerr et al. (2016) have highlighted the beneficial effects of taking genetic structure into account when independently estimating the biomass of such stock components.

Norwegian offshore and coastal components of cod have been studied for decades (Nordeide, Johansen, Jørgensen, Karlsen, & Moum, 2011). The offshore and highly migratory Barents Sea cod (also called north‐east Arctic cod or north‐east Atlantic cod, abbreviated to NEAC) is the world's largest remaining cod stock (Garrod & Schumacher, 1994; Yaragina, Aglen, & Sokolov, 2011). NEAC spawn in Norwegian coastal waters during the spring, from Møre (62°N) in the south to the Russian border in the north (Olsen et al., 2010). Eggs and larvae resulting from NEAC spawning drift northwards to the Barents Sea where they grow and feed until maturity (>7 years), when they migrate back to the Norwegian coast to spawn (Brander, 1995; Johansen et al., 2017; Olsen et al., 2004). In contrast to long‐migrating NEAC, Norwegian coastal cod are relatively stationary and are found on the Norwegian shelf and coastline and within fjords, throughout the year (Jakobsen, 1987; Michalsen, Johansen, Subbey, & Beck, 2014).

**FIGURE 1:**
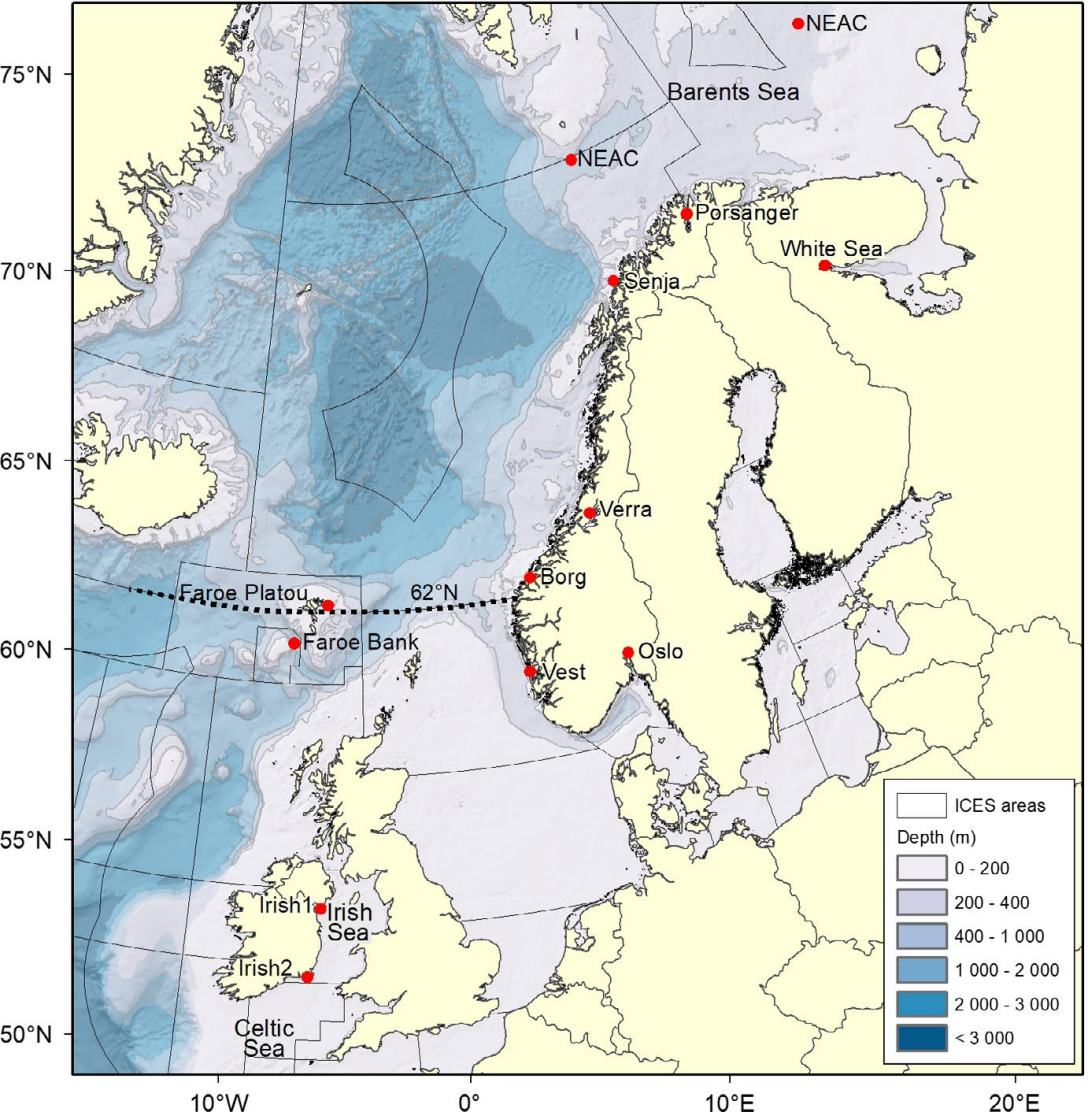
Study area including sampling sites

Norwegian coastal cod are currently managed as two separate units: north (Norwegian coastal cod: NCC) and south (Norwegian coastal south: NCS) of 62°N (Figure 1). The management of NCS is linked to the assessment of the North Sea cod (ICES, 2012). NCC have historically been exposed to a higher harvest pressure than NCS, and approximately 70% of the NCC harvest occurs during the spring spawning season (Johansen et al., 2017). Both NCC and NCS fisheries are presently regarded to be outside sustainable harvest limits and therefore in need of a thorough management revision (ICES, 2012, 2018). For both NCC and NCS, spawning typically occurs within fjords and outer skerries, and the cod captured here have traditionally been described as Fjord or Bank cod (Nordeide et al., 2011).

Population genetics of coastal cod in Norwegian waters have been investigated, both in the northern (NCC: Skarstein, Westgaard, & Fevolden, 2007) and in the southern regions (NCS: Knutsen, Jorde, André, & Stenseth, 2003; Knutsen et al., 2011), as well as along the entire Norwegian coastline (Dahle et al., 2018). The latter study, including an analysis of >4,000 cod from 55 spawning sites, revealed a pattern of genetic isolation by distance along the Norwegian coastline without any clearly defined population boundaries. Furthermore, introgression with NEAC, which followed a decreasing N‐S latitudinal pattern to almost none south of 62°N, contributed to the overall pattern in genetic structure for NCC. In the southern part of the NCS range, that is Norwegian Skagerrak, several studies have shown low but statistically significant differences between fjord populations (Jorde, Knutsen H, Espeland, & Stenseth, 2007; Knutsen et al., 2003; Sodeland et al., 2016). The observed level of genetic divergence in this region is consistent with data from tagging studies showing low levels of dispersal (Knutsen et al., 2011) and geographic fine‐scale variation in life‐history traits of coastal cod (Olsen et al., 2004).

A failure to correctly define genetic stocks is illustrated by the historical and current annual quota agreement formulated by Norway and Russia for coastal cod, where NCC are added to the 5–10 times larger quota of NEAC (ICES, 2019; Jakobsen, 1987). From the mid‐1970s until 2003, an expected annual catch of 25, 000 tonnes for Norwegian and Russian NCC was set within this combined NEAC/NCC quota. ICES provides management advice for NEAC and NCC in the Barents Sea and Norwegian Russian management areas, and the total quota has been driven primarily by the status of the NEAC stock, thus leading to an inherent risk of overexploiting the smaller NCC stock. Due to the decline of NCC, ICES advised a zero catch of NCC for the years 2004–2011 supplemented by a recovery plan (ICES, 2015, 2019).

The genomic resources available for Atlantic cod have rapidly expanded in recent years, for example, by the development of genome assemblies (Star et al., 2011; Tørresen et al., 2017) and a SNP array (Berg et al., 2016). These tools have permitted, for example, distinguishing between migratory and nonmigratory ecotypes throughout the species range (Barney, Munkholm, Walt, & Palumbi, 2017; Berg et al., 2017; Berg et al., 2016; Bradbury et al., 2013; Kirubakaran et al., 2016). Between these two ecotypes, distinct islands of divergence have been observed in linkage groups (LGs) 1, 2, 7 and 12 (Berg et al., 2016; Sodeland et al., 2016). The island of divergence on LG1 coincides with a double inversion (Berg et al., 2017; Kirubakaran et al., 2016). Inversions on LG12 seem to differentiate between NCS and the North Sea cod in the south of Norway (Barth et al., 2017; Sodeland et al., 2016), whereas in the Gulf of Maine, islands of divergence on LG2, LG7 and LG12 allow to identify the three spawning units in this region potentially linked with local adaptation (Barney et al., 2017).

Despite recent advances in our understanding of population structure in Atlantic cod, many questions still remain and can be addressed with genomic data to help advise management of cod populations. We used the recently developed SNP array to analyse population genetic structure in coastal cod from multiple locations of coastal cod in Norway presently managed as two stocks north and south of 62°N. In addition, samples from White Sea, Ireland and Faroe Islands were included to provide a wider framework for interpreting genomic variation within and among cod stock components.

## 2 MATERIALS AND METHODS

### 2.1 Sample collection, DNA extraction and genotyping

All the Norwegian and White Sea cod included in the present study (Table 1) were sampled as a part of a large sampling programme funded by the Institute of Marine Research in Norway (IMR) by gillnets or by longline. Cod representing two Irish samples (landings north‐east and south of Ireland) and the Faroe Islands (two samples; landing from Faroe Plateau and from offshore Faroe Bank cod) were taken from commercial trawlers. Gill tissue was stored in 96% ethanol prior to DNA extraction. Genomic DNA was extracted using the Qiagen DNeasy Blood & Tissue Kit (Qiagen, Germany). DNA quality and quantity were assessed by agarose gel electrophoresis and Qubit fluorometry (Thermo Fisher Scientific, USA). Taken together, twelve geographic locations were represented by 549 individuals (Figure 1; Table 1).

**TABLE 1:** Samples information: *N* corresponds to the initial number of individuals screened, whereas Nf corresponds to the numbers of individuals used for analysis once NEAC (based on otolith reading), and badly amplified samples were removed

Management areas	Site	*N*	Nf	Sex (% F)	Age range (year)	Maturity stage (1–5)	Spawning fish
Barents Sea	NEAC	48	47	0.4	1–8	1–5	0.13
White Sea	White Sea	48	47	0.73	2–4	2–5	1.00
NCC (north of 62°N)	Porsangerfjord	48	39	0.23	5–11	2–4	1.00
	Senja	48	42	0.42	1–13	1–2	0.13
	Verrabotn	48	37	0.13	3–9	2–4	1.00
	Borgundfjord	48	45	0.77	4–10	2–4	1.00
NSC (south of 62°N)	Vest	48	48	0.69	2–9	1–3	0.92
	Oslo	48	44	0.56	2–12	1–5	0.79
Outgroups	Faroe Bank	40	40	NA	NA	NA	NA
	Faroe Plateau	29	29	–	–	–	–
	Irish 1	48	20	–	–	–	–
	Irish 2	48	48	–	–	–	–

Note

Sex ratio, age and maturity (in years) are indicated as well as the proportion of spawning fish.

Otoliths from the Norwegian samples were read to determine the age of the fish and to classify them as NEAC or Norwegian coastal cod (NCC/NCS). Otolith types 1 and 2 depict coastal cod, whereas 4 and 5 are assigned to NEAC, according to Rollefsen (1933) and Berg and Albert (2003). Both the NCC and NCS show similar otolith patterns. Individual cod with otolith category NEAC were removed from the NCC samples to exclude any migratory NEAC (this meant removing 16 individuals in total from the northern samples).

All samples were genotyped using a custom Illumina SNP array containing assays for 10,913 SNPs (Berg et al., 2016) according to the manufacturer's instructions (Illumina, San Diego, USA). Individuals displaying a call rate below 0.9 were excluded from analyses as were nonpolymorphic SNPs or SNP with a call rate <0.95, leaving a final data set of 486 fish analysed with 8,174 genome‐distributed SNPs.

### 2.2 Outlier detection

In large marine populations, most of the genetic markers might be uninformative about demographic structure (Ward, Woodwark, & Skibinski, 1994), so loci carrying signature of local divergence are useful to outline management units for fisheries management (Russello, Kirk, Frazer, & Askey, 2012). To stratify the SNP data set into loci nondeviating from neutrality and candidates to selection, outlier analyses were conducted using three analytical approaches: BayeScan (Foll & Gaggiotti, 2008), LOSITAN (Antao, Lopes, Lopes, Beja‐Pereira, & Luikart, 2008) and PCAdapt (Luu, Bazin, & Blum, 2017). BayeScan v.2.1 (Foll & Gaggiotti, 2008) was used with default parameters, and the log^10^(BF)>0.5 criterion, that is “substantial” evidence for selection according to Jeffreys (1961), was chosen to define non‐neutral markers. In LOSITAN, a neutral distribution of *F*_ST_ with 1,000,000 iterations was simulated, with forced mean *F*_ST_ at a significance level of 0.05 under an infinite allele model. It has been suggested that outlier tests may produce high false‐positive rates because of population demography and bottlenecks (see, e.g., Lotterhos & Whitlock 2014; Narum & Hess 2011; de Villemereuil & Gaggiotti 2015). A way to circumvent this problem is to conduct the analyses between pairs of populations, since this partly overlooks the methodological weakness of population structure/demographic processes (Vitalis, Dawson, & Boursot, 2001). Hence, a first analytical step consisted of pooling all the samples to detect SNPs under selection across all geographic regions. Secondly, outlier scans were individually performed for each pair of samples to determine whether the selection pressure changed across locations or whether it reflected any geographic pattern. Markers were regarded as neutral if they were categorized as such in all pairs of samples, and they were considered as outliers if they were found to be under positive selection in, at least, one pair of samples. SNPs under balancing selection, identified by negative alpha value both from BayeScan and from LOSITAN graphic output, were found in extremely low numbers and discarded from this study. The computationally demanding pairwise tests were performed only with BayeScan.

**FIGURE 2:**
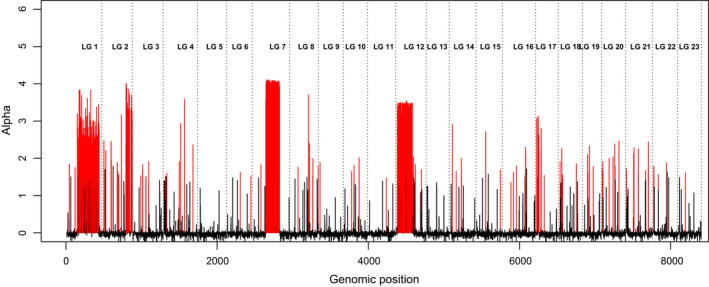
Outlier scan. SNPs are ordered according to their positions on each of the 23 LGs. The estimated alpha coefficient indicates the strength and direction of selection, being positive for diversifying and negative for balancing. The alpha reported on the y‐axis: dark lines for *p* > .001 and red lines for *p* < .001

Consensus genome scan of outlier detection revealed large portions of LG1, LG2, LG7 and LG12 to be under likely positive selection (see Figure 2 and Figure S1a,b). Therefore, the data set was subsequently stratified into six sub data sets for population structure analyses (two neutral and four non‐neutral ones). Two sets of putative neutral markers were defined: one containing loci outside LG1, LG2, LG7 and LG12, which was named “Neutrals‐A” (*n* = 5,854 SNPs), and a second one containing SNPs on LG1, LG2, LG7 and LG12, but outside the regions of these chromosomes that were deemed to be under positive selection, which was called “Neutrals‐B” (*n* = 1,344). A further four sets of candidate markers to positive selection were defined within the selected areas on LG1 (LG1S, *n* = 281), LG2 (LG2S; *n* = 75), LG7 (LG7S; *n* = 185) and LG12 (LG12S; *n* = 200), respectively.

### 2.3 Genetic clustering

STRUCTURE v.2.3.4 (Pritchard, Stephens, & Donnelly, 2000) was run separately on the six subsets of data, as defined above, under a model assuming admixture and correlated allele frequencies but without using population information. For each data set, ten runs, with a burn‐in period consisting of 100,000 replications and a run length of 1,000,000 Markov chain Monte Carlo (MCMC) iterations, were performed for *K* = 1 to *K* = 8 clusters. STRUCTURE runs were automatized with the program ParallelStructure (Besnier & Glover 2013) to reduce computational time. Output was analysed using two approaches: the ad hoc summary statistic Δ*K* from Evanno, Regnaut, and Goudet (2005), which detects the uppermost hierarchical level of structure in the data, and using the four statistics (MedMed, MedMean, MaxMed and MaxMean) implemented in StructureSelector (Li & Liu, 2018). The latter has been described as more accurate than the previously used methods to determine the best‐fit number of clusters, for both even and uneven sampling data. Finally, runs for the selected Ks were averaged with CLUMPP v.1.1.1 (Jakobsson & Rosenberg 2007) using the FullSearch algorithm and the G’ pairwise matrix similarity statistic and were graphically displayed using bar plots.

Genetic clustering was investigated with all six data sets by discriminant analysis of principal components (DAPC) (Jombart, Devillard, & Balloux, 2010), in which the coordinates of all the individuals were calculated on a set of discriminant axes aiming to maximize the variance between region and to minimize the variance within regions. Plotting all the individuals on a two‐dimensional space consisting of the two first discriminant axes provided a graphical representation of the genetic distance and relative positions of the samples. The DAPC was performed in R (R Core Team, 2018), using the adegenet package (Jombart, 2008; Jombart & Ahmed, 2011).

### 2.4 Haplotype reconstruction

The large regions under positive selection in LG1, LG2, LG7 and LG12 were characterized by high levels of linkage disequilibrium. Using a 15‐SNP sliding window, extended haplotypes for each LG were reconstructed separately for the twelve sampling locations using the software PHASE v2.1 (Stephens, Smith, & Donnelly, 2004). Some of those haplotypes were represented in high frequency in all, or most of, the sampling sites. The frequency of the haplotypes was reported in all sampling sites for any sequence of 15‐SNP haplotype that reached the minimum frequency of 10% in at least one sampling site. The frequency of each haplotype per site was plotted on a map obtained from the R packages “maps” (Becker & Wilks, 2018) and “mapplots” (Gerritsen, 2018).

### 2.5 Genetic distances and Mantel test

Pairwise genetic distances (*F*_ST_) between sampling sites were computed separately for each of the six subsets of SNPs defined above using Weir and Cockerham (1984) unbiased estimator implemented in the software Arlequin v.3.5.1.2 (Excoffier, Laval, and Schneider, 2005). Statistical significance for the null hypothesis of no genetic divergence was assessed by 10,000 permutations.

The relationship between genetic and geographic distances was explored by testing whether the genetic data fitted a (linear) pattern of isolation by distance (IBD). A two‐tailed Mantel (1967) test was conducted between the matrices of genetic distance (estimated as pairwise *F*_ST_) and geographic distance (defined as the shortest marine path between sites and measured using Google Earth). The analyses were conducted using PaSSaGE v.2 (Rosenberg & Anderson, 2011), and 10,000 permutations were used to calculate the significance of the correlations.

The slopes of IBD tests allow to obtain qualitative estimates of mean dispersal distances using the theoretical model elaborated by Kinlan and Gaines (2003) and based on Palumbi (2003); i.e. dispersal distance = 0.0016(IBD slope)^−1.0001^. The average dispersal distance obtained for markers under positive selection was compared with the distance for neutral loci to assess whether gene flow might be influenced by any form of environmental pressure.

### 2.6 Detection of loci associated with environmental variables

The identification of candidate SNPs putatively involved in local adaptation was addressed with two complementary approaches. First, outlier analysis was used to flag loci most strongly associated with the observed population structure, which was located within LG1, LG2, LG7 and LG12 (see above). Then, LFMM, “latent factor mixed model” (Frichot, Schoville, Bouchard, & François, 2013), was used to identify loci showing unusual associations with environmental variables compared to the genetic background. LFMM accounts for the underlying population structure by introducing latent factors while simultaneously estimating random effects driven by isolation by distance and population history. The environmental factor tested was temperature, measured at 50 m depth, in the months of March and July as they are assumed to be important for spawning and juvenile growth, respectively. Hence, associations between genetic variation of loci belonging to LG1, LG2, LG7 and LG12 and temperature were assessed while controlling for neutral genetic structure with (random) latent factors. Ten runs of LFMM were conducted using 1,000 sweeps for burn‐in and 10,000 additional sweeps. The number of latent factors was set at *K* = 4 according to STRUCTURE as suggested by Frichot et al. (2013). As the variation among runs was extremely low, only the first one was kept. Significance was chosen after Bonferroni correction for multiple tests.

## 3 RESULTS

### 3.1 Assessing non‐neutral genomic regions of divergence

The initial scan for outlier detection performed across all of the 23 linkage groups (LGs) in all of the samples (8,174 SNPs) revealed that LG1, LG2, LG7 and LG12 contained relatively large genomic regions deviating from neutrality, as indicated by a large number of SNPs scoring a high probability for being under positive selection (Figure 2 and Figures S1a,bandS2). Conversely, other genomic regions showed less evidence of selection.

### 3.2 Genetic clustering

**FIGURE 3:**
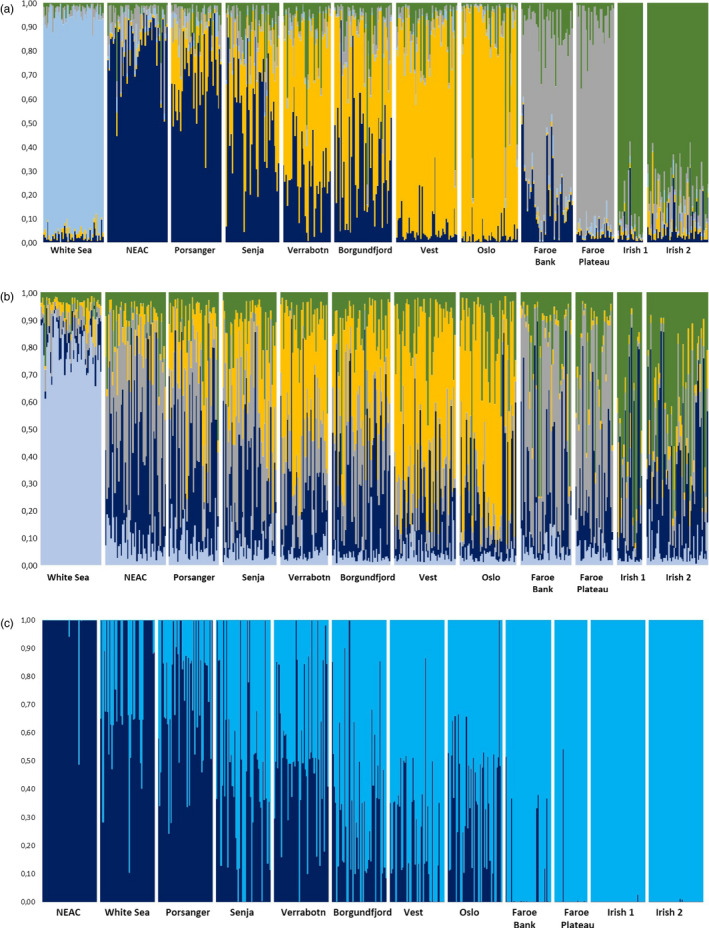
Bayesian clustering of the twelve samples with: (a) 5,854 neutral SNPs (Neutrals‐A), (b) 1,433 neutral SNPs within the four LG groups (Neutrals‐B), and (c) the pools of SNPs identified as under positive selection on LG1, LG2, LG7 and LG12 when including NEAC. Inferred ancestry of individuals was calculated after averaging ten STRUCTURE runs with CLUMPP; see Table S1 for Evanno test and StructureSelector results

Evanno test revealed a major division at *K* = 2 (Table S1) for both sets of neutral markers. The highest differentiation was observed between the White Sea and the remaining samples at *K* = 2 (Figure S3a). Conversely, five genetic groups were identified by StructureSelector (Table S1) for all neutral markers. In the Neutrals‐A data set, the genetic clustering pattern somehow revealed a geographic underlying pattern (Figure 3a); that is White Sea, Vest‐Oslo, Faroe samples, Irish samples and a gradient from NEAC to Borgundfjord in the remaining NCC samples. The clustering pattern was more diffuse in the data set using the neutral SNPs located within the LG, the White Sea being the only distinct group (Figure 3b). The candidate SNPs to positive selection located within LG1, LG2, LG7 and LG12 combined revealed a gradient in which the NCC samples in the northernmost region of Norway were genetically closer to the NEAC (Figure 3c, more details in Figure S4a–k). By removing the NEAC from the plot, the White Sea sample stands out as the most deviating sample (plot not shown).

**FIGURE 4:**
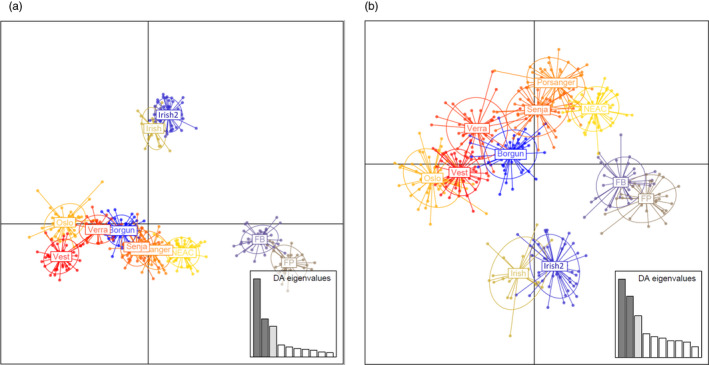
Discriminant analyses of principal components (DAPC) of the samples from eleven sites (i.e. excluding the White Sea) based on neutral markers: (a) 5,854 SNPs (Neutrals‐A) and (b) 1,344 SNPs (Neutrals‐B). Plots containing the White Sea can be found in Figure S5a‐b in Supplementary Information

**FIGURE 5:**
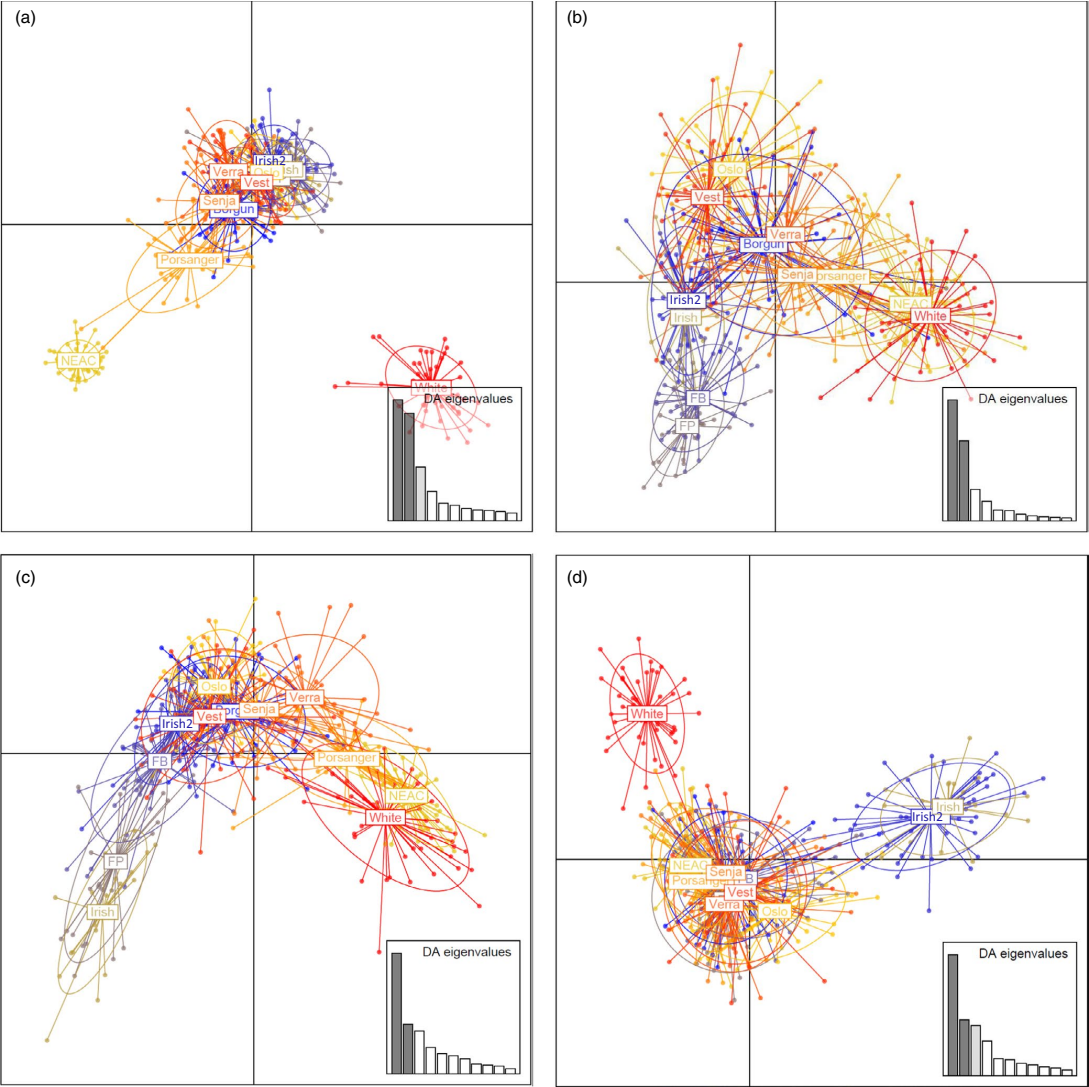
Discriminant analyses of principal components (DAPC) of the samples from the twelve sites based on SNPs under positive selection on: (a) LG1, (b) LG2, (c) LG7 and (d) LG12

The DAPC outcome for the sets A and B of neutral markers (Figure 4a‐b: including the White Sea; Figure S5a,b) agreed with STRUCTURE, with samples from different countries clustering separately while a gradient of genetic similarity was revealed from north to south in Norwegian coastal cod. However, the patterns revealed by the SNPs under selection varied among the four LGs (Figure 5a‐d). For instance, within LG1, the axis corresponding to the first component (PC1) defined three pools: NEAC, White Sea and the remaining samples (Figure 5a), whereas for LG12 (Figure 5d), the three pools distinctly separated by PC1 were as follows: White Sea, the Irish samples and the rest. While the LG2 (Figure 5b) and LG7 (Figure 5c) show latitudinal gradients in the Norwegian samples, the NEAC and White Sea samples are overlapping.

### 3.3 Geographic patterns in reconstructed haplotypes

Haplotypes were constructed for each sample along 15‐SNP sliding windows for each of the four LGs displaying markers under selection (Table 2, Figure S6). For each LG, one or several haplotypes were observed in moderate to high frequencies in all the samples. For example, in LG1 (Figure S6), the 15‐SNP haplotype starting at SNP position 223 was found in moderate to high frequencies ranging from 24% in Oslo up to 96% in NEAC (Table 2). Interestingly, this region of highly shared haplotypes was very narrow around SNP no. 223 in all samples, apart from NEAC, where the highly shared haplotypes seemed to cover a large portion of the chromosome (between SNP nos. 120 and 400, Figure S6). Similarly, in LG2, LG7 and LG12, at least one locus presented a haplotype with a high frequency in all or most of the regions (Table 2).

**TABLE 2:** Frequencies of the most common haplotype (%) within each geographic sample

LG	SNP frame	Frequency of the most common haplotype (%)											
		White Sea	NEAC	NCC				NCS		Faroe Islands		Ireland	
				Porsanger	Senja	Verrabotn	Borgundfjord	Vest	Oslo	Bank	Plateau	Irish 1	Irish 2
LG1	223–237	31	96	35	30	35	27	28	24	39	33	30	36
LG2	337–351	40	52	34	33	22	25	20	24	12	11	17	16
LG7	205–219	61	91	78	41	59	25	20	24	18	18	42	22
LG7	252–266	25	20	18	51	42	71	78	65	82	85	83	95
LG7	287–301	70	85	78	54	55	65	77	68	85	96	97	94
LG7	330–344	45	62	59	56	42	72	78	70	87	97	99	96
LG12	29–43	88	83	79	70	66	79	50	32	59	52	45	38

Note

**FIGURE 6:**
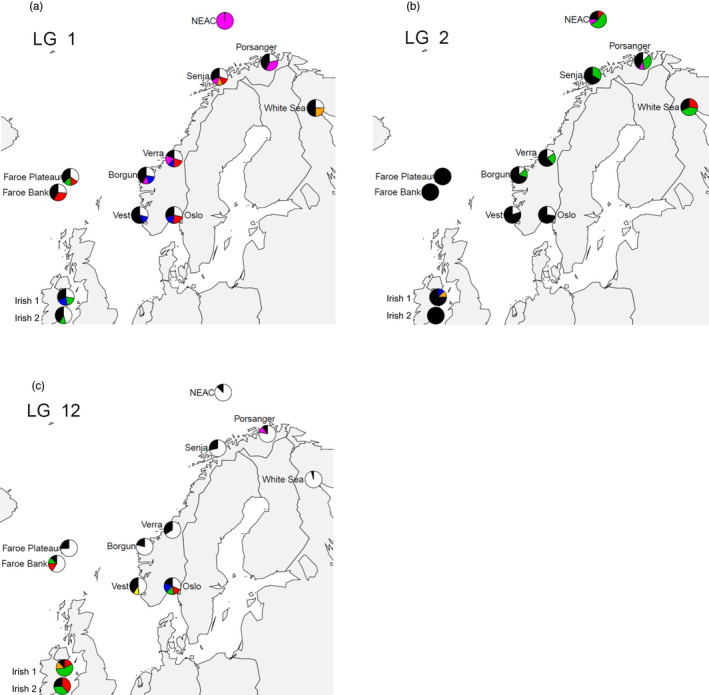
Geographic distribution of haplotypes: (a) LG1: (b) LG2: (c) LG12: geographic repartition of the haplotypes with frequency ≥ 10% in at least one region. Each haplotype is identified by the same given colour across sampling sites. Black represents the sum of all other haplotypes displaying < 10% frequencies. (i.e. all frequencies always sum to 100%)

**FIGURE 7:**
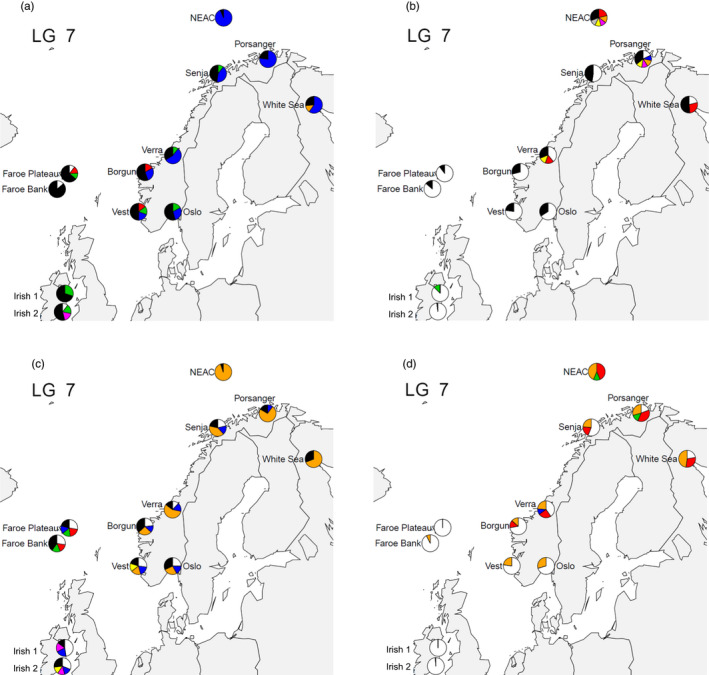
Geographic distribution haplotypes. The four plots represent different subregions of LG7 (cf. Table 2). (a) LG7 SNP frame 205–219, (b) LG7 SNP frame 252–266, (c) LG7 SNP frame 287–301 and (d) LG7 SNP frame 330–344. Each haplotype is identified by the same given colour across sampling sites. Black represents the sum of all other haplotypes displaying < 10% frequencies

One row represents one genomic region or “locus,” defined by linkage groups (LG) and SNP frame (or numbers) within LG, and may contain different haplotypes in different samples. See also Figures 6 and 7 and text for details.

For each genomic position where a haplotype was reported (see Table 2), haplotypes present in ≥ 10% of the individuals in at least one sampling site were visualized in Figures 6, 7. In LG1, the frequency of the one haplotype was almost fixed in the NEAC sample (Pink: Figure 6a), but gradually decreased in frequency southwards in the NCC to finally disappear in the two NCS sites. A second haplotype (white) was present in all samples but NEAC, whereas the blue haplotype was shared by the coastal cod from Verra (NCC) to Oslofjord (NCS). The White Sea sample shared haplotypes (black/white) with the NCC and NCS except for the yellow haplotype also found in the one NCC from Senja.

In LG2, haplotypes were less conserved than in LG1 (Figure 6b). In some regions (Faroe Bank and Irish samples), all haplotypes were present at frequencies less than 10% (shown in black). One haplotype (in red) was only present in the White Sea and NEAC samples. Haplotype in green was mostly represented in the NCC, NEAC and White Sea samples, and the frequency gradually decreased southwards, whereas the white haplotype was present in >28% of the Oslo (NCS) sample and gradually decreased in frequency towards the north.

In LG12, haplotype 1 (white) was the most common in all northern samples between White Sea and Vest, but completely absent from the Irish samples. In the southern samples, haplotypes in green and red were present in high frequencies in the NCS sample from Oslofjord, but completely absent in NCC, NEAC and White Sea samples (Figure 6c).

LG7 displayed several genomic regions where haplotypes were found in high frequencies (Table 2). The geographic distribution of haplotypes varied greatly from one locus to another on LG7, which is illustrated in four different haplotype maps (Figure 7a‐d). The first 15‐SNP haplotype that spanned from SNP 205 to SNP 219 on LG7 was almost fixed in NEAC, that is the same sequence of 15 SNPs was found in all individuals from the NEAC sample (Figure 7a), whereas the NCC samples displayed a higher haplotype diversity. The same pattern was found in the haplotype that spanned from SNP 287 to 301 (Figure 7c). The two other loci (SNP252‐266 and 330–344) displayed a higher haplotype diversity with 3–6 distinct SNP sequences that were found both in the NEAC and NCC samples (Figure 7b, d). NCC and NCS are more variable in Figure 7a, c, while unique regional haplotypes were found in NCC (Figure 7d).

### 3.4 Genetic differentiation and Isolation by distance

Global *F*_ST_ for neutral loci was low, albeit statistically significant: 0.007 (*p* < .0001) for Neutrals‐A and 0.010 (*p* < .0001) for Neutrals‐B. Following expectations, global *F*_ST_ for loci under positive selection within the different LGs ranged from 0.122 to 0.253 (*p* < .0001). Pairwise *F*_ST_ matrices for both sets of neutral loci can be found in Table 3 and in Table S2a–d for each LG. At the neutral loci, the White Sea deviated highly from the remaining samples including NCC and NCS. The samples within NCC were significantly different from the NCS for both sets of neutral markers, but no differentiation was found within NCC by neutral SNPs. The average degree of differentiation between NEAC and any of the other samples was 0.62, whereas the average *F*_ST_ of the remaining comparisons was 0.02 (28‐fold difference).

**TABLE 3:** *F*_ST_ values between pairs of samples calculated with ARLEQUIN based on Neutrals‐A data set (below diagonal) and Neutrals‐B (above diagonal)

	White Sea	NEAC	NCC				NCS		Faroe Islands		Ireland	
			Porsanger	Senja	Verrabotn	Borgundfjord	Vest	Oslo	Bank	Plateau	Irish 1	Irish 2
White Sea		**0.022**	**0.024**	**0.028**	**0.038**	**0.034**	**0.034**	**0.037**	**0.021**	**0.030**	**0.026**	**0.028**
NEAC	**0.019**		0.002	**0.002**	**0.007**	**0.004**	**0.008**	**0.011**	**0.004**	**0.004**	0.000	**0.005**
Porsanger	**0.021**	0.001		0.001	**0.004**	**0.004**	**0.005**	**0.007**	**0.009**	**0.009**	0.000	**0.008**
Senja	**0.023**	0.002	0.000		**0.003**	0.001	**0.004**	**0.007**	**0.006**	**0.006**	0.000	**0.006**
Verrabotn	**0.026**	**0.003**	**0.003**	0.000		0.001	**0.006**	**0.007**	**0.014**	**0.015**	**0.001**	**0.008**
Borgundfjord	**0.021**	0.002	0.002	0.000	0.000		**0.003**	**0.008**	**0.012**	**0.013**	0.000	**0.004**
Vest	**0.028**	**0.008**	**0.008**	**0.003**	**0.003**	**0.002**		**0.003**	**0.016**	**0.016**	0.000	**0.008**
Oslo	**0.027**	**0.009**	**0.006**	**0.003**	0.001	**0.003**	0.000		**0.014**	**0.016**	0.001	**0.012**
Faroe Bank	**0.022**	**0.003**	**0.003**	**0.006**	**0.006**	**0.003**	**0.011**	**0.010**		**0.004**	0.000	**0.009**
Faroe Plateau	**0.026**	**0.003**	**0.005**	**0.005**	**0.006**	**0.008**	**0.010**	**0.010**	0.000		**0.005**	**0.011**
Irish 1	**0.025**	0.002	**0.004**	**0.004**	**0.004**	0.000	**0.008**	**0.005**	0.002	**0.005**		0.000
Irish 2	**0.021**	**0.003**	**0.003**	**0.002**	**0.002**	**0.002**	**0.006**	**0.007**	**0.004**	**0.003**	0.001	

Note

Boldface type depicts values significantly different from zero at *α* = 0.05 (after 10,000 permutations).

A series of two‐tailed Mantel tests were conducted for each set of SNPs in different subsets of sampling sites to assess the correlation between geographic and genetic distance. The total suite of twelve sites significantly followed IBD expectations for all the set of loci (range from *M* = 0.439–0.662, *p* = .033–.000) except the markers under selection within LG1 (*m* = 0.136, *p* = .160; but see Table S3). When excluding the White Sea from the analyses, the IBD pattern was lost from the neutral markers but present in the four sets of outliers. The third series of tests, conducted only with Norwegian coastal cod from the management units NCC and NCS, showed a relatively strong IBD in all data sets (Neutral‐A: *m* = 0.657, *p* = .024; Neutral‐B: *m* = 0.550, *p* = .039; LG1: *m* = 0.732, *p* = .002; LG12; m = 0.572, *p* = .043) except for LG2 (0.369, *p* = .120) and LG7 (*m* = 0.502, *p* = .064). The dispersal distance estimated for neutral markers, when considering the full set of samples was 320 km, whereas the dispersal for loci under selection within LG was significantly smaller (<50 km).

### 3.5 Detection of loci associated with environmental variables

Loci showing significant association with sea temperature in March were mostly found within LG7, followed by LG12 (91% and 80%, respectively) conversely to the 34% of the loci in LG1 and 55% in LG2. In all cases, percentages slightly increased when testing for sea temperature in July. However, in terms of the strength of the association, LG7 singled out in both comparisons. By setting the threshold of ‐log_10_(PO)=15, 69% of the loci in LG7 showed association with temperature in March and 84% in July. Among the rest of the linkage groups, the highest value was 6% of loci overcoming this threshold in July at LG12 (see Figure S7a,b).

## 4 DISCUSSION

This is the first study to investigate genetic structure of Norwegian coastal cod across the two management units using a population genomics SNP approach. We observed pairwise genetic differences between all six coastal cod populations, from Porsanger fjord in the far north to Oslo fjord in the south‐east. Although the degree of differentiation among samples depended on the set of markers being considered, population genetic differences were observed in all four genomic regions under selection (i.e. LG1, LG2, LG7 and LG12), as well as in both sets of neutral markers (i.e. Neutrals‐A and Neutrals‐B). The present ICES management regime for coastal cod in Norway is formulated around the 62°N latitude divide, that is Norwegian coastal cod is at present managed as two stocks. Our data demonstrate that while the present management division at 62°N does capture break in gene flow to the south of this border, it does not sufficiently represent genetic structuring in the north and is in need of revision. In order to aid in defining further management boundaries for coastal cod, finer geographic sampling is needed.

Norwegian coastal cod spawn in sheltered fjords and more open coastal areas (Jakobsen, 1987). Although spawning varies greatly in time and space (Johansen et al., 2017; Otterå, Agnalt, & Jørstad, 2006; Otterå et al., 2018), NEAC and NCC overlap on the spawning grounds in some areas. Depending on the markers considered, genetic differentiation observed herein largely followed a north‐to‐south gradient of similarity with the NEAC sample, whereby the sample from Oslo fjord was the most differentiated to NEAC, and the sample from Porsanger fjord was the least differentiated to NEAC. This gradient is apparent from the population structuring analysis (STRUCTURE, DAPC and IBD: Figures 3, 4, 5, Figures S3–S4, respectively). A similar trend of genetic isolation by distance merging towards NEAC in the north has previously been observed (Dahle et al., 2018). Collectively, these studies demonstrate a combination of mixing and gene flow between NEAC and coastal cod that follows a north‐to‐south gradient, thus driving at least partly the observed population genetic structure. Previous studies have demonstrated gene flow between NEAC and NCC (Berg et al., 2016; Rodríguez‐Ramilo et al., 2019), but not its spatial pattern.

The observed population genetic structure of Norwegian coastal cod could have arisen by different mechanisms but ultimately implies spatially restricted dispersal and gene flow. Taken at face value, the results of the Mantel tests indicate a dispersal distance of Norwegian coastal cod of some 500–800 km (Table S3). Although these estimates should be treated with caution, they align somewhat to the observed migratory distance of NCC that has been estimated to be up to ~300 km on the basis of results from tagging experiments (Michalsen et al., 2014). NCC also show spawning site fidelity (Jakobsen, 1987; Michalsen et al., 2014). The observed gradient of relatedness to NEAC may then have arisen in situ since the postglacial colonization of the coastline by a combination of genetic drift and limited dispersal distance. Another, more likely mechanism is that coastal and NEAC cod would have already diverged prior to colonization and the gradient developed through secondary contact. Such a hypothesis has been suggested when studying population divergence in flatfish from the Baltic and the North Sea as demographic history revealed that the age of the Baltic lineage was actually older than the Baltic Sea itself (Le Moan et al., 2019). Within the latter interpretation, NCC seems more introgressed with NEAC than NCS, a pattern that has some tentative support from the Structure analyses of neutral variation (Figure 3).

### 4.1 Patterns in non‐neutral SNPs and reconstructed haplotypes

Regions with putative selected genes coincided with the four previously known chromosome inversions on LG1, LG2, LG7 and LG12. Spatial patterns of genetic variation at these regions revealed potentially useful information regarding population structuring in Norwegian coastal cod and adjacent waters. It appears that each genomic region reflects a different pattern. Briefly, LG1 largely distinguishes NEAC and White Sea from the rest (cf. Figure 5a): one single haplotype dominates the whole NEAC sample (96%), while the same haplotype is present in other samples along the Norwegian coast in a decreasing gradient of frequency towards the south (Figure 6a). This pattern appears to reflect a gradient of introgression of NEAC into NCC from north to south. In the southern populations (the Irish Sea and Faroe), other haplotypes are present in high frequency, indicating additional selective forces operating at the same “locus” at LG1 independently from the influence of NEAC.

Selected SNP at LG2 and LG7 regions largely coincided with the neutral ones in describing a pattern of genetic differentiation that followed geographic positions (Figure 5b‐c), while also give indication of positive selection between most pairs of samples. The most novel pattern was observed in LG7, where at least four regions or “loci” (starting at SNP positions 205, 252, 287 and 330) appeared to be carrying highly frequent haplotypes across several regions. Representing the frequency of the common haplotypes on each sample (Figure 7a‐d) also revealed that each locus reflects a different pattern. Haplotypes starting on SNP position 205 (Figure 7a) and 287 (Figure 7c) are characterized by one unique haplotype present in >80% of the NEAC samples and an increasing gradient of haplotype variety towards the south. On SNP positions 252 (Figure 7b) and 330 (Figure 7d), we observe the opposite phenomenon, with a unique haplotype dominating most of the southern sample with frequency >75% and an increasing gradient of haplotype diversification towards the north. This could reflect different adaptation to temperature in the different areas (Clucas et al., 2019). Of all the LG we tested for local adaptation, only LG7 was linked to temperature in March and July reflecting spawning and larval growth (Figure S7a,b). More detailed investigation of the specific SNP is warranted future research.

Finally, LG12 separated from all others the White Sea and southern samples (cf Figure 5d) while also showed positive selection in the latter. LG12 has previously been found to differentiate between North Sea and coastal cod in the southern Norway (Sodeland et al., 2016).

### 4.2 White Sea cod

This is the first study to compare the genetics of White Sea cod with neighbouring Norwegian coastal cod. Cod from the White Sea are unique as they are the only coastal cod population inhabiting the Arctic waters of the North Atlantic. Although its life history is poorly understood, recent studies have suggested it to be resident in the White Sea and spawn under the ice at water temperatures as cold as −1.8°C (Makhotin, 2016; Yershov, Marschal, Ereskovsky, & Vishnyakov, 2016). Earlier, White Sea cod has been ascribed to a subspecies due to its divergent biology (*Gadus morhua marisalbi:* Derjugin 1920), and genetic analyses using mtDNA polymorphisms have confirmed its recent divergence from *Gadus morhua* (Zelenina, Makeenko, Volkov, & Mugue, 2016). Here, we detected highly significant genetic differences between the White Sea cod and Norwegian coastal cod, and between the White Sea cod and all other samples. These differences were highly distinct. For example, pairwise *F*_ST_ values between the White Sea sample and all others using the Neutral‐A and Neutral‐B sets of SNPs gave values ranging from 0.019 to 0.028 and 0.021 to 0.038, respectively (Table 3). In contrast, pairwise *F*_ST_ values between all other population pairs excluding the White Sea sample ranged from 0 to 0.011 and 0 to 0.016, respectively. The White Sea sample also displayed differentiation in the markers under selection on LG1, LG2, LG7 and LG12. Collectively, these analyses demonstrate that White Sea cod are highly divergent to other cod analysed and thus that this population have been isolated longer if not a subspecies.

### 4.3 The outgroup samples: Ireland and Faroe Islands

Our analysis demonstrated that samples from Ireland and Faroe Islands were distinct from each other and from all the Norwegian samples including NEAC. The Faroe Island cod stocks divided into the Faroe Island Plateau cod and the Faroe Bank cod showed weak divergence between the two, supporting a hypothesis of panmixia in this region. The cod from Faroe bank together with the cod from the Celtic Sea are among the fastest growing cod (Magnussen, 2007). Microsatellites have found low but significant genetic difference between the Bank and the Faroe Plateau cod (Nielsen, Wright, et al., 2009). The Faroe Bank cod have shown resemblance to the NEAC cod in haemoglobin polymorphism (Fyhn, Brix, Nævdal, & Johansen, 1994) but did not show any such association in the present study. The Bank cod experience higher temperature than cod on the Faroe Plateau (Magnússon, Bergstad, Hareide, Magnússon, & Reinert, 1997), which is higher than that experienced by the Norwegian coastal cod and NEAC. The Faroe Bank cod mature later and at larger size than cod on the Faroe Plateau (Brander, 1995). In spite of these differences in life history, only subtle differences were found between them by our SNP panels. The SNP markers included on the cod SNP array (Berg et al., 2016) were identified in samples collected from a broad geographic distribution of Norwegian waters. Nevertheless, the inversions located in LG7 and LG12 were present for these outgroup samples and do indicate differentiation in these stocks that needs to be characterized.

## 5 CONCLUSIONS

We have revealed a complex genetic variability within coastal cod. This variability is not currently been reflected in the management plan, with its potential consequences for depletion of genetic diversity. The current dividing of Norwegian coastal cod into only two management units, north (NCC) and south (NCS) of 62°N, is not sufficient to reflect the true biological units and needs to be revised. Although 62°N can be the natural border between north and southern populations, most likely there is an even finer structure both north and south of this border. It will be challenging to devise an optimal management strategy that adequately reflects the patterns of genetic variability within coastal cod. The apparent north–south cline in large parts of the genome does not easily lend itself to interpretation in distinct units, and much more geographic fine‐scaled approach seems necessary to resolve genetic units. There are still issues to look further into, as the potential areas under selection, that could maybe help in changes in management plan in the future.

This study has shown that coastal cod in Norway, while highly heterogenous, is also genetically distinct from neighbouring stocks in the north (NEAC, White Sea), west (Faroe Island) and the south (Irish samples). We further found that the White Sea cod are highly divergent from other cod, possibly yielding support the earlier notion of subspecies rank. The other two outlier samples were clearly different from the Norwegian coastal cod and NEAC but showed only subtle differences within, which could be influenced by ascertainment bias.

## Supporting information

Appendix S1

## Data Availability

Data for this study are available at: available on request from lead author and in supplement.
