# Supplementary material for: Genomic analysis reveals neutral and adaptive patterns that challenge the current management regime for East Atlantic cod Gadus morhua L
Source: Evol Appl. 2020 Sep 5;13(10):2673–88. doi: 10.1111/eva.13070 (PMC7691467; doi:10.1111/eva.13070)
Supplement: Supplementary file 1 — Appendix S1 [file EVA-13-2673-s001.pdf]

SUPPLEMENTARY INFORMATION

Table S1.- Analyses of STRUCTURE outputs following Evanno test and StructureSelector for the neutral markers and the loci under selection:

		Evanno test						StructureSelector			
**Dataset**	**K**	**Runs**	**Mean LnP(K)**	**Stdev LnP(K)**	**Ln'(K)**	**|Ln''(K)|**	**ΔK**	**MedMedK**	**MedMeaK**	**MaxMedK**	**MaxMeaK**
**Neutrals_A**	1	10	-3007270.96	4.97	NA	NA	NA	5	5	6	6
	2	10	-2997201.05	16.23	10069.91	4438.68	**273.45**				
	3	10	-2991569.82	72.03	5631.23	4928.61	68.42				
	4	10	-2990867.2	452.92	702.62	439.30	0.97				
	5	10	-2990603.88	560.66	263.32	32306.39	57.62				
	6	10	-3022646.95	110858.74	-32043.07	65431.78	0.59				
	7	10	-2989258.24	1194.36	33388.71	38654.91	32.36				
	8	10	-2994524.44	5773.30	-5266.20	NA	NA				
**Neutrals_B**	1	10	-678314.9	1.06	NA	NA	NA	4	4	5	5
	2	10	-675973.3	7.86	2341.60	1772.76	**225.47**				
	3	10	-675404.46	89.15	568.84	439.18	4.93				
	4	10	-674396.44	103.80	1008.02	1365.01	13.15				
	5	10	-674753.43	190.66	-356.99	2382.44	12.50				
	6	10	-677492.86	5555.54	-2739.43	681.00	0.12				
	7	10	-679551.29	4043.54	-2058.43	4742.27	1.17				
	8	10	-676867.45	2798.26	2683.84	NA	NA				
**LG1**	1	10	-153469.1	0.26	NA	NA	NA	5	5	5	5
	2	10	-128718.84	0.26	24750.26	22850.94	**88207.77**				
	3	10	-126819.52	2.27	1899.32	1430.22	628.97				
	4	10	-126350.42	5.27	469.10	205.35	38.97				
	5	10	-126086.67	9.54	263.75	1176.31	123.27				
	6	10	-126999.23	2470.56	-912.56	2379.87	0.96				
	7	10	-125531.92	15.90	1467.31	1927.56	121.25				
	8	10	-125992.17	1516.26	-460.25	NA	NA				

Dataset	K	Runs	Mean LnP(K)	Stdev LnP(K)	Ln'(K)	|Ln''(K)|	ΔK	MedMedK	MedMeaK	MaxMedK	MaxMeaK
**LG2**	1	10	-41111.44	0.10	NA	NA	NA	3	3	4	4
	2	10	-29557.22	0.43	11554.22	11155.34	**26006.02**				
	3	10	-29158.34	200.68	398.88	50.60	0.25				
	4	10	-28810.06	299.99	348.28	285.60	0.95				
	5	10	-28747.38	219.97	62.68	74.02	0.34				
	6	10	-28758.72	237.47	-11.34	39.57	0.17				
	7	10	-28730.49	216.95	28.23	60.48	0.28				
	8	10	-28762.74	306.49	-32.25	NA	NA				
**LG7**	1	10	-110174.48	0.23	NA	NA	NA	3	3	3	3
	2	10	-50226.82	0.23	59947.66	59880.53	**255073.92**				
	3	10	-50159.69	20.19	67.13	35.08	1.74				
	4	10	-50127.64	44.57	32.05	1.04	0.02				
	5	10	-50094.55	68.12	33.09	53.81	0.79				
	6	10	-50115.27	137.73	-20.72	23.42	0.17				
	7	10	-50112.57	133.36	2.70	27.41	0.21				
	8	10	-50137.28	206.89	-24.71	NA	NA				
**LG12**	1	10	-116318.31	0.27	NA	NA	NA	3	3	3	3
	2	10	-59101.36	0.44	57216.95	57029.58	**131064.97**				
	3	10	-58913.99	132.72	187.37	6.22	0.05				
	4	10	-58720.4	103.43	193.59	110.31	1.07				
	5	10	-58637.12	75.85	83.28	6.57	0.09				
	6	10	-58560.41	77.66	76.71	306.51	3.95				
	7	10	-58790.21	351.04	-229.80	40.86	0.12				
	8	10	-59060.87	843.99	-270.66	NA	NA				
**Pool selected**	1	10	-306802,31	0,24	NA	NA	NA	4	4	4	4
	2	10	-222628,5	0,54	84173,81	46038,28	**84683,64**				
	3	10	-184492,97	0,72	38135,53	28444,50	39696,44				
	4	10	-174801,94	5048,80	9691,03	8669,23	1,72				
	5	10	-173780,14	5894,60	1021,80	5236,94	0,89				
	6	2	-167521,4	171,97	6258,74	NA	NA				

Table S2.- Average (over SNPs) pairwise F_{ST} between populations calculated with ARLEQUIN (lower diagonal) and P-values after 10000 permutations (upper diagonal) for six different datasets. P-values highlighted in boldface type are statistically significant at α=0.05.

a) Loci under positive selection within **LG1** (the original dataset was composed by N=281 SNPs, but ARLEQUIN dismissed 3 loci):

	**White Sea**	**NEAC**	**Porsanger**	**Senja**	**Verrabotn**	**Borgundfjord**	**Vest**	**Oslo**	**Faroe Bank**	**Faroe Plateau**	**Irish 1**	**Irish 2**
**White Sea**		**0.0000**	**0.0000**	**0.0000**	**0.0000**	**0.0000**	**0.0000**	**0.0000**	**0.0000**	**0.0000**	**0.0000**	**0.0000**
**NEAC**	0.6201		**0.0000**	**0.0000**	**0.0000**	**0.0000**	**0.0000**	**0.0000**	**0.0000**	**0.0000**	**0.0000**	**0.0000**
**Porsanger**	0.0792	0.4760		**0.0056**	**0.0005**	**0.0004**	**0.0000**	**0.0000**	**0.0000**	**0.0000**	**0.0004**	**0.0000**
**Senja**	0.0485	0.5652	0.0205		0.1837	0.4196	**0.0003**	**0.0000**	**0.0006**	**0.0006**	**0.0493**	**0.0001**
**Verrabotn**	0.0536	0.6255	0.0367	0.0011		0.5936	0.1103	**0.0058**	**0.0001**	**0.0000**	0.0520	**0.0000**
**Borgundfjord**	0.0443	0.5821	0.0313	0.0000	0.0000		**0.0084**	**0.0102**	**0.0003**	**0.0011**	0.1085	**0.0006**
**Vest**	0.0465	0.6250	0.0626	0.0099	0.0015	0.0046		0.7322	**0.0000**	**0.0000**	0.1521	**0.0000**
**Oslo**	0.0523	0.6350	0.0655	0.0111	0.0043	0.0051	0.0000		**0.0000**	**0.0000**	**0.0245**	**0.0008**
**Faroe Bank**	0.0463	0.6266	0.0572	0.0135	0.0113	0.0124	0.0136	0.0114		0.1269	**0.0097**	**0.0000**
**Faroe Plateau**	0.0506	0.6815	0.0645	0.0138	0.0134	0.0103	0.0143	0.0131	0.0023		**0.0057**	**0.0007**
**Irish 1**	0.0451	0.7455	0.0591	0.0052	0.0022	0.0024	0.0012	0.0038	0.0055	0.0059		0.6554
**Irish 2**	0.0426	0.6222	0.0647	0.0114	0.0107	0.0071	0.0059	0.0053	0.0102	0.0069	0.0000	

b) Loci under positive selection within **LG2** (the original dataset was composed by N=75 SNPs, but ARLEQUIN dismissed 3 loci):

	**White Sea**	**NEAC**	**Porsanger**	**Senja**	**Verrabotn**	**Borgundfjord**	**Vest**	**Oslo**	**Faroe Bank**	**Faroe Plateau**	**Irish 1**	**Irish 2**
**White Sea**		**0.0000**	**0.0003**	**0.0000**	**0.0000**	**0.0000**	**0.0000**	**0.0000**	**0.0000**	**0.0000**	**0.0000**	**0.0000**
**NEAC**	0.0252		**0.0000**	**0.0000**	**0.0000**	**0.0000**	**0.0000**	**0.0000**	**0.0000**	**0.0000**	**0.0000**	**0.0000**
**Porsanger**	0.0516	0.0871		**0.0039**	**0.0392**	**0.0000**	**0.0000**	**0.0001**	**0.0000**	**0.0000**	**0.0000**	**0.0000**
**Senja**	0.1701	0.2417	0.0478		**0.0439**	0.3878	**0.0009**	**0.0309**	**0.0000**	**0.0000**	**0.0000**	**0.0000**
**Verrabotn**	0.1179	0.1726	0.0134	0.0163		**0.0135**	**0.0000**	**0.0159**	**0.0000**	**0.0000**	**0.0000**	**0.0000**
**Borgundfjord**	0.2021	0.2733	0.0674	0.0000	0.0207		**0.0046**	0.1056	**0.0000**	**0.0000**	**0.0000**	**0.0000**
**Vest**	0.3322	0.4028	0.1721	0.0494	0.0910	0.0268		**0.0076**	**0.0000**	**0.0000**	**0.0004**	**0.0000**
**Oslo**	0.2238	0.2876	0.0808	0.0224	0.0229	0.0079	0.0302		**0.0000**	**0.0000**	**0.0000**	**0.0000**
**Faroe Bank**	0.4989	0.5762	0.3495	0.1765	0.2754	0.1766	0.1225	0.2202		0.1218	**0.0000**	**0.0000**
**Faroe Plateau**	0.5249	0.6056	0.3723	0.1980	0.2959	0.1955	0.1414	0.2413	0.0038		**0.0000**	**0.0000**
**Irish 1**	0.5163	0.6034	0.3359	0.1521	0.2462	0.1299	0.0608	0.1675	0.0343	0.0609		0.9054
**Irish 2**	0.5066	0.5799	0.3495	0.1733	0.2626	0.1505	0.0713	0.1798	0.0547	0.0803	0.0000	

c) Loci under positive selection within **LG7** (the original dataset was composed by N=185 SNPs, but ARLEQUIN dismissed 2 loci):

	**White Sea**	**NEAC**	**Porsanger**	**Senja**	**Verrabotn**	**Borgundfjord**	**Vest**	**Oslo**	**Faroe Bank**	**Faroe Plateau**	**Irish 1**	**Irish 2**
**White Sea**		**0.0000**	0.1674	**0.0000**	**0.0059**	**0.0000**	**0.0000**	**0.0000**	**0.0000**	**0.0000**	**0.0000**	**0.0000**
**NEAC**	0.1555		**0.0000**	**0.0000**	**0.0000**	**0.0000**	**0.0000**	**0.0000**	**0.0000**	**0.0000**	**0.0000**	**0.0000**
**Porsanger**	0.0075	0.0940		**0.0000**	**0.0001**	**0.0000**	**0.0000**	**0.0000**	**0.0000**	**0.0000**	**0.0000**	**0.0000**
**Senja**	0.1530	0.4585	0.2184		0.0852	**0.0348**	**0.0006**	**0.0264**	**0.0000**	**0.0000**	**0.0000**	**0.0000**
**Verrabotn**	0.0590	0.3538	0.1082	0.0174		**0.0001**	**0.0000**	**0.0002**	**0.0000**	**0.0000**	**0.0000**	**0.0000**
**Borgundfjord**	0.3065	0.6084	0.3822	0.0322	0.1271		0.2004	0.6278	**0.0006**	**0.0000**	**0.0006**	**0.0000**
**Vest**	0.4016	0.6882	0.4800	0.0919	0.2174	0.0061		0.0941	**0.0060**	**0.0002**	**0.0023**	**0.0000**
**Oslo**	0.2980	0.6004	0.3737	0.0295	0.1211	0.0000	0.0092		**0.0000**	**0.0000**	**0.0001**	**0.0000**
**Faroe Bank**	0.5623	0.8275	0.6458	0.2427	0.4012	0.1063	0.0485	0.1117		**0.0337**	**0.0388**	**0.0000**
**Faroe Plateau**	0.6185	0.8822	0.7049	0.3153	0.4762	0.1777	0.1144	0.1844	0.0205		**0.0003**	**0.0000**
**Irish 1**	0.6006	0.8819	0.6884	0.2932	0.4529	0.1610	0.0984	0.1664	0.0316	0.0440		0.9096
**Irish 2**	0.6577	0.8876	0.7369	0.3579	0.5224	0.2048	0.1304	0.2130	0.0511	0.0519	0.0000	

d) Loci under positive selection within **LG12** (the original dataset was composed by N=200 SNPs, but ARLEQUIN dismissed 5 loci):

	**White Sea**	**NEAC**	**Porsanger**	**Senja**	**Verrabotn**	**Borgundfjord**	**Vest**	**Oslo**	**Faroe Bank**	**Faroe Plateau**	**Irish 1**	**Irish 2**
**White Sea**		**0.0000**	**0.0000**	**0.0001**	**0.0000**	**0.0000**	**0.0000**	**0.0000**	**0.0000**	**0.0000**	**0.0000**	**0.0000**
**NEAC**	0.0275		**0.0045**	**0.0001**	**0.0000**	**0.0011**	**0.0000**	**0.0000**	**0.0000**	**0.0031**	**0.0000**	**0.0000**
**Porsanger**	0.0410	0.0180		0.1334	**0.0266**	0.3994	**0.0000**	**0.0000**	**0.0001**	0.2452	**0.0000**	**0.0000**
**Senja**	0.0784	0.0589	0.0073		0.4761	0.8098	**0.0012**	**0.0000**	**0.0236**	0.3902	**0.0000**	**0.0000**
**Verrabotn**	0.1152	0.0958	0.0269	0.0000		0.2213	**0.0087**	**0.0000**	0.1018	0.1788	**0.0000**	**0.0000**
**Borgundfjord**	0.0601	0.0397	0.0000	0.0000	0.0055		**0.0000**	**0.0000**	**0.0055**	0.4395	**0.0000**	**0.0000**
**Vest**	0.2702	0.2561	0.1585	0.0887	0.0581	0.1170		**0.0029**	0.0836	**0.0005**	**0.0000**	**0.0000**
**Oslo**	0.5032	0.4884	0.3827	0.2955	0.2526	0.3341	0.0749		**0.0000**	**0.0000**	**0.0003**	**0.0000**
**Faroe Bank**	0.1874	0.1700	0.0842	0.0316	0.0155	0.0529	0.0171	0.1630		**0.0162**	**0.0000**	**0.0000**
**Faroe Plateau**	0.0734	0.0413	0.0028	0.0004	0.0117	0.0000	0.1252	0.3418	0.0537		**0.0000**	**0.0000**
**Irish 1**	0.8159	0.7975	0.7085	0.6177	0.5878	0.6529	0.3625	0.1657	0.4764	0.6910		0.4261
**Irish 2**	0.7685	0.7551	0.6781	0.5989	0.5689	0.6311	0.3511	0.1437	0.4644	0.6580	0.0000	

Table S3.- Two-tailed Mantel tests comparing matrices of shortest-water geographic distance in Km (GeoDistance) and genetic distance measured as pairwise F_{ST} for the following dataset: Neutral markers set A, Neutral markers set B and markers under divergent selection in LG1.2.7 and 12. Numbers in bold depict significant values after 9999 permutations. *Norwegian coastal cod excludes White Sea, NEAC and the outlier samples from Ireland and Faroe Island.

Geographic range	Matrices	Mantel's r	P-value	Dispersal distance (km)
All twelve sites	GeoDistance x NeutralsA-F_{ST}	0.52426	**0.00650**	320
	GeoDistance x NeutralsB-F_{ST}	0.43908	**0.03340**	320
	GeoDistance x LG1-DirF_{ST}	0.13614	0.16060	53
	GeoDistance x LG2-DirF_{ST}	0.66290	**0.00040**	16
	GeoDistance x LG7-DirF_{ST}	0.62893	**0.00050**	8
	GeoDistance x LG12-DirF_{ST}	0.59821	**0.00090**	8
All sites excluding White Sea	GeoDistance x NeutralsA-F_{ST}	0.06357	0.35230	2289
	GeoDistance x NeutralsB-F_{ST}	0.02770	0.45160	8012
	GeoDistance x LG1-DirF_{ST}	0.28092	**0.04780**	18
	GeoDistance x LG2-DirF_{ST}	0.59998	**0.00110**	16
	GeoDistance x LG7-DirF_{ST}	0.66496	**0.00030**	8
	GeoDistance x LG12-DirF_{ST}	0.63131	**0.00060**	8
Norwegian coastal cod*	GeoDistance x NeutralsA-F_{ST}	0.65701	**0.02420**	534
	GeoDistance x NeutralsB-F_{ST}	0.55003	**0.03940**	801
	GeoDistance x LG1-DirF_{ST}	0.73240	**0.00290**	53
	GeoDistance x LG2-DirF_{ST}	0.36999	0.12000	53
	GeoDistance x LG7-DirF_{ST}	0.50213	0.06490	16
	GeoDistance x LG12-DirF_{ST}	0.57283	**0.04350**	16

a)

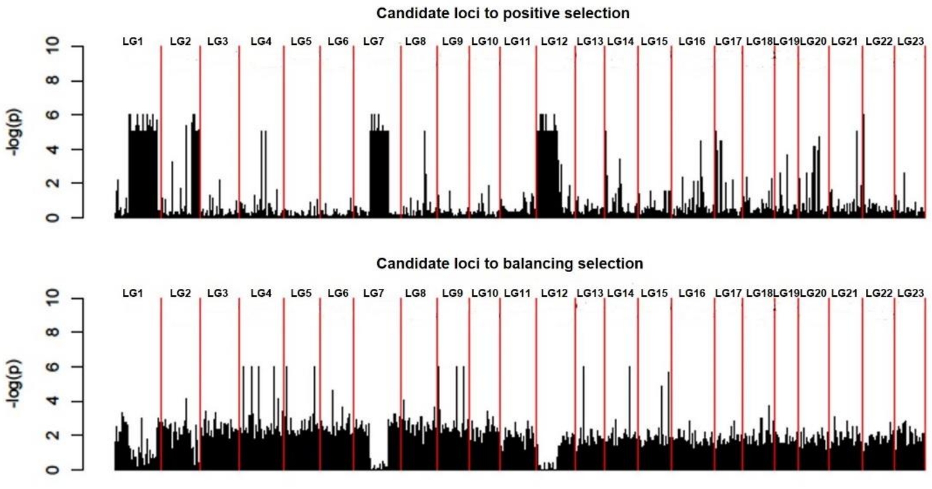

b)

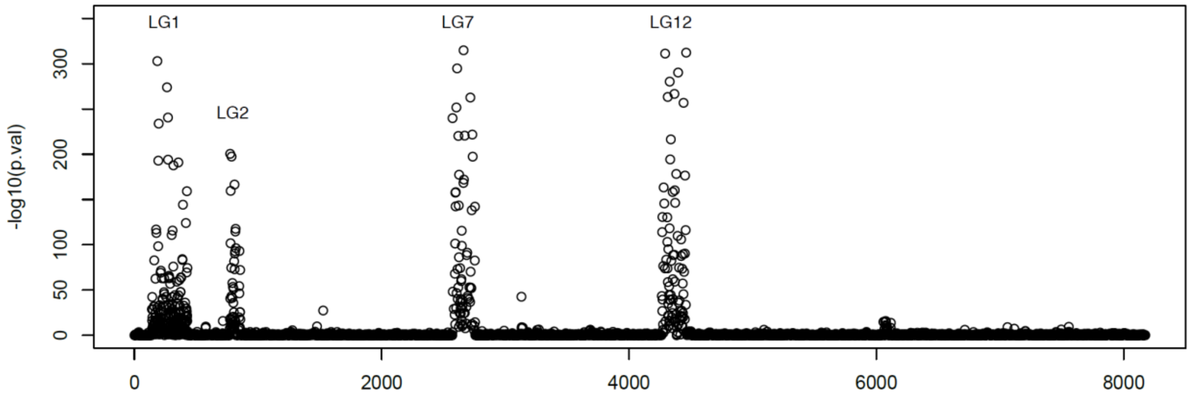

Figure S1. Outlier scan. SNPs are ordered according to their positions on each of the 23 LG's. The estimated alpha coefficient indicates the strength and direction of selection, being positive for diversifying and negative for balancing. a) LOSITAN b) PCAdapt.

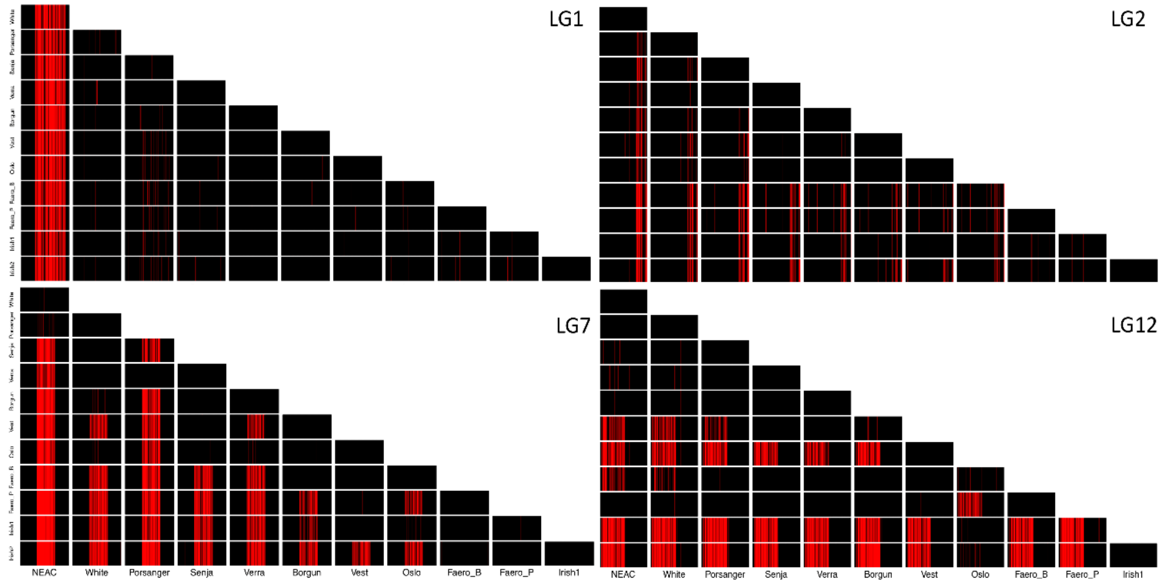

Figure S2. LG1, LG2, LG7 and LG12 plots: Lower diagonal matrix where each sub-plot is a scan for markers under selection between two geographic areas. Every SNP in the four LGs is represented by a vertical bar: the black ones depicting neutral ones. and the red ones depicting SNPs under positive selection. as judged by the outlier tests.

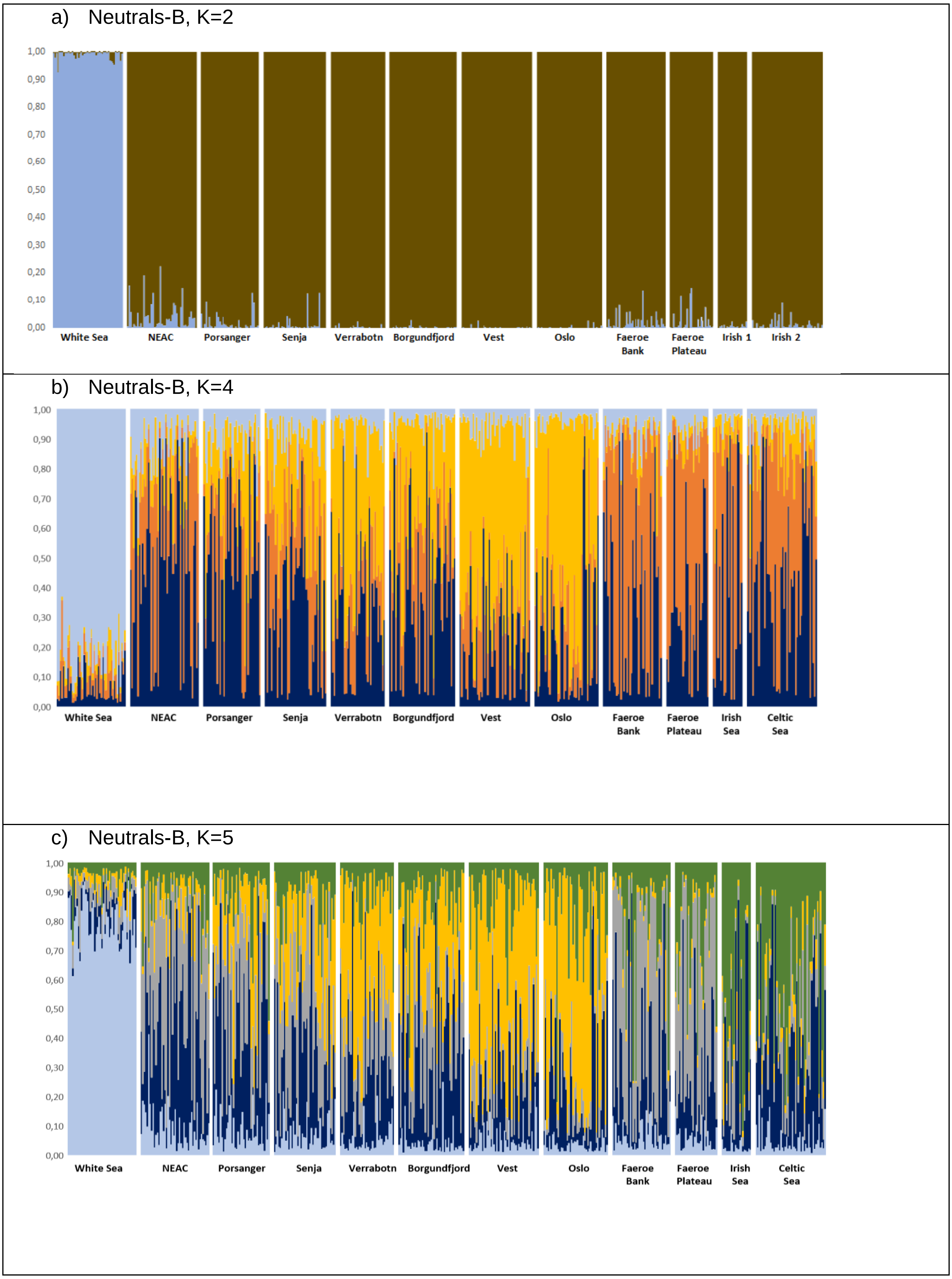

Figure S3. Bayesian clustering for the twelve populations genotyped at Neutrals-B loci. Inferred ancestry of individuals was calculated after averaging ten STRUCTURE runs with CLUMPP. See Table S1 for Evanno test and StructureSelector results.

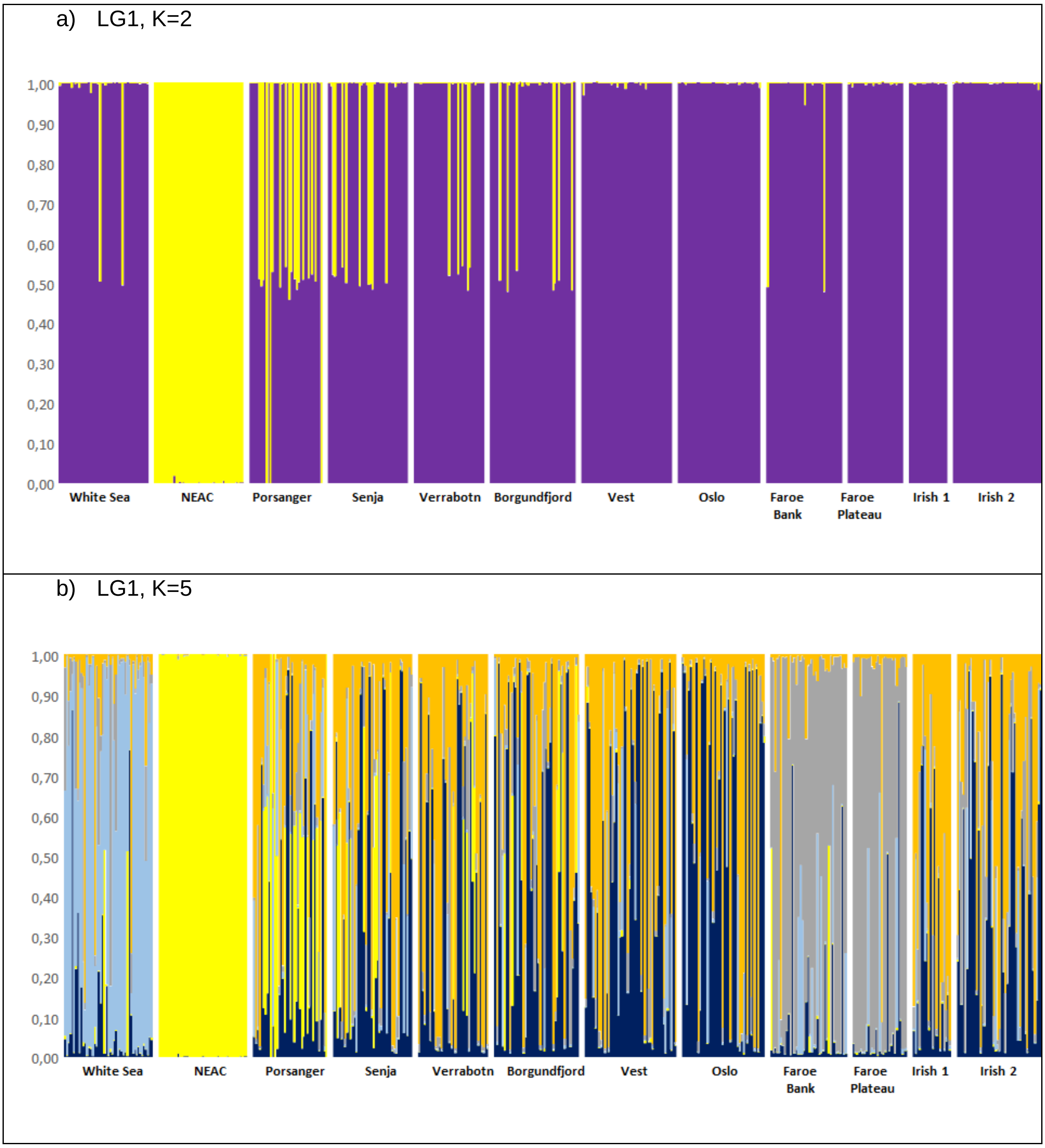

a) LG1, K=2
1,00
0,90
0,80
0,70
0,60
0,50
0,40
0,30
0,20
0,10
0,00
White Sea
NEAC
Porsanger
Senja
Verrabotn
Borgundfjord
Vest
Oslo
Faroe Bank
Faroe Plateau
Irish 1
Irish 2
b) LG1, K=5
1,00
0,90
0,80
0,70
0,60
0,50
0,40
0,30
0,20
0,10
0,00
White Sea
NEAC
Porsanger
Senja
Verrabotn
Borgundfjord
Vest
Oslo
Faroe Bank
Faroe Plateau
Irish 1
Irish 2

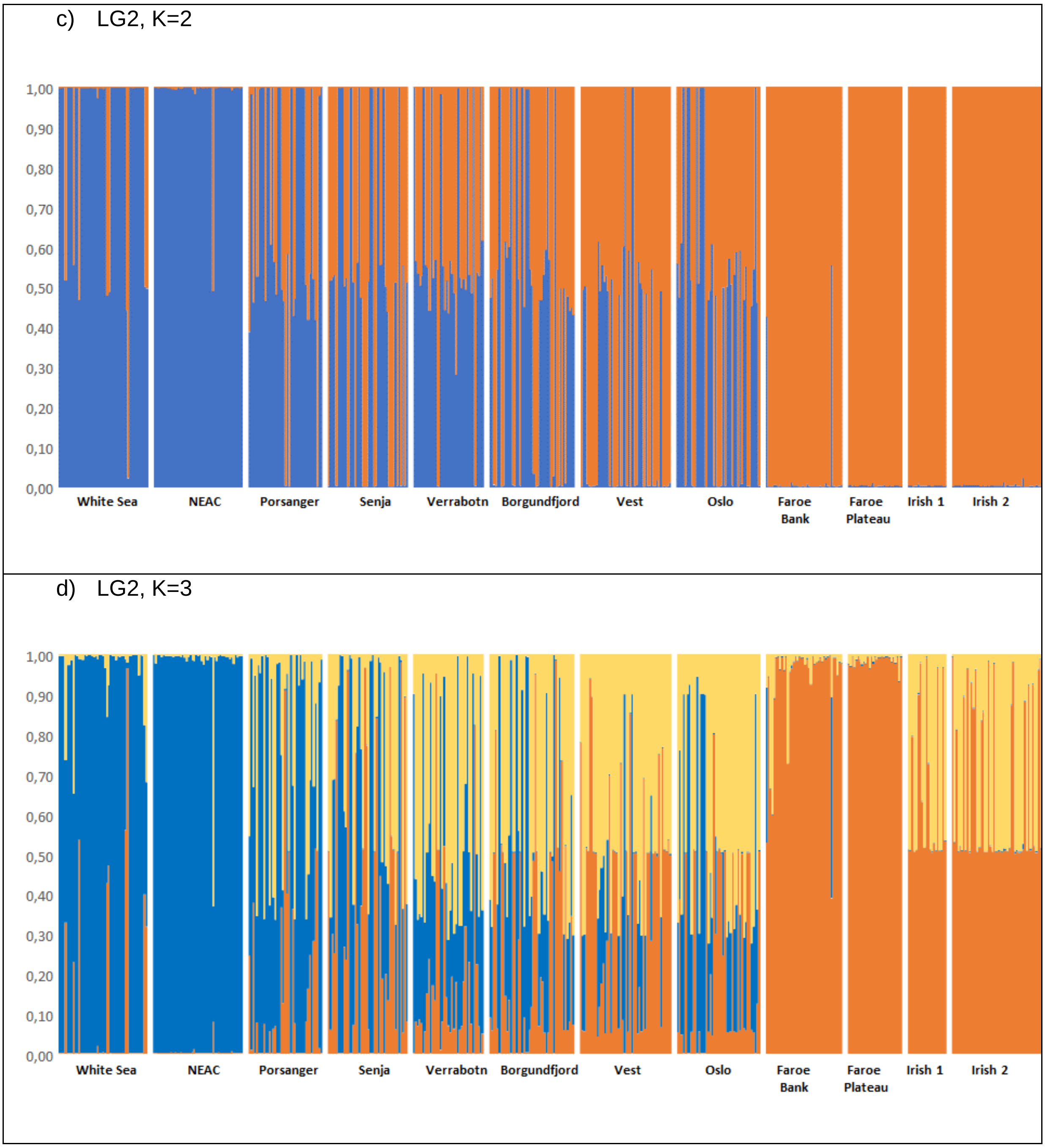
c) LG2, K=2
1,00
0,90
0,80
0,70
0,60
0,50
0,40
0,30
0,20
0,10
0,00
White Sea
NEAC
Porsanger
Senja
Verrabotn
Borgundfjord
Vest
Oslo
Faroe Bank
Faroe Plateau
Irish 1
Irish 2
d) LG2, K=3
1,00
0,90
0,80
0,70
0,60
0,50
0,40
0,30
0,20
0,10
0,00
White Sea
NEAC
Porsanger
Senja
Verrabotn
Borgundfjord
Vest
Oslo
Faroe Bank
Faroe Plateau
Irish 1
Irish 2

e) LG2, K=4

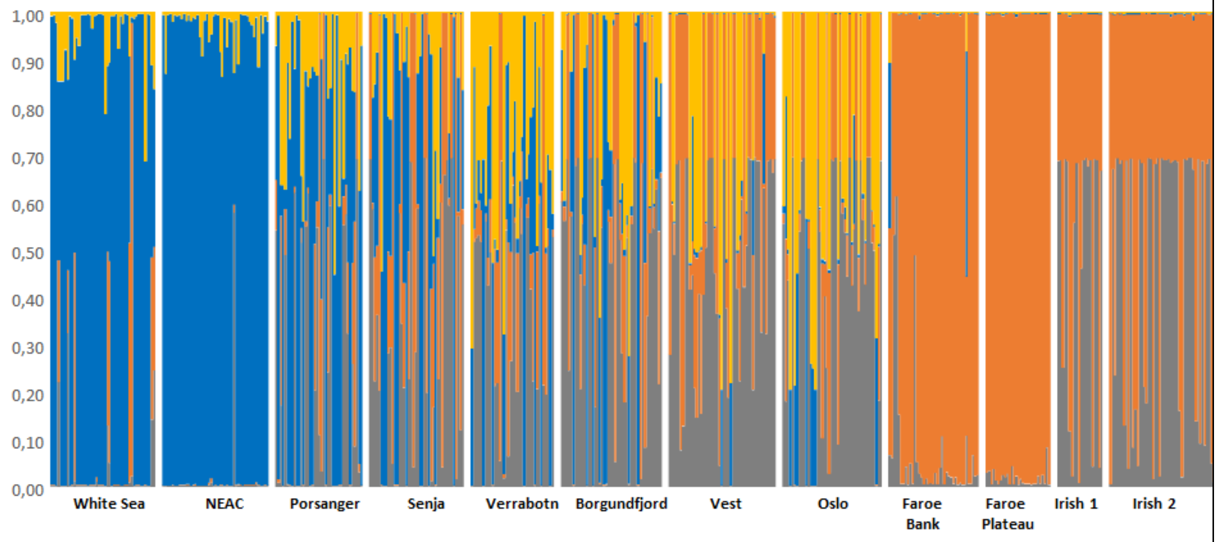

f) LG7, K=2

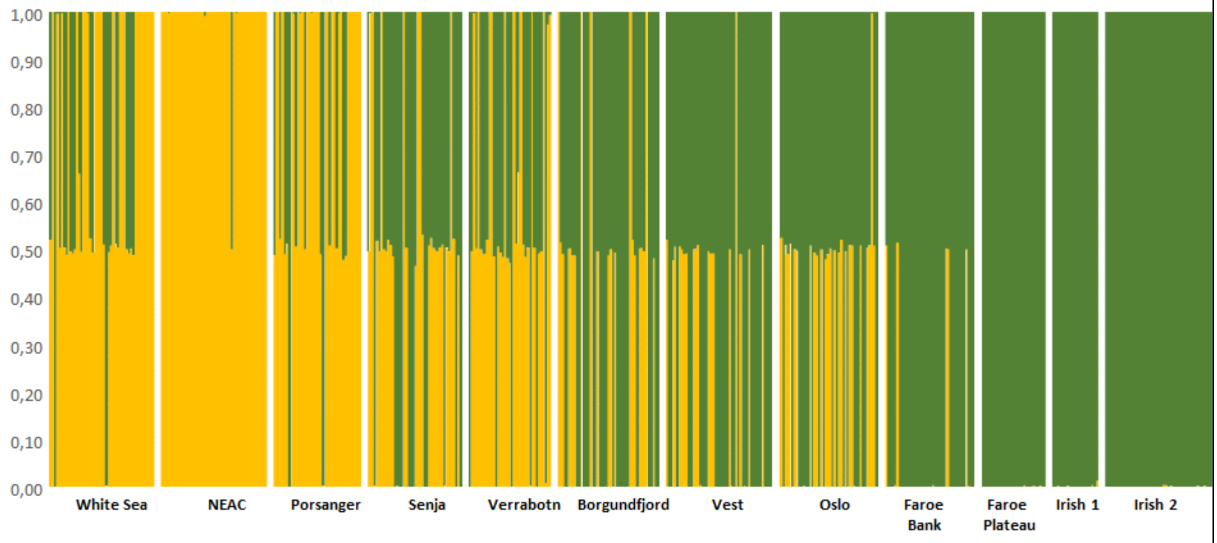

g) LG7, K=3

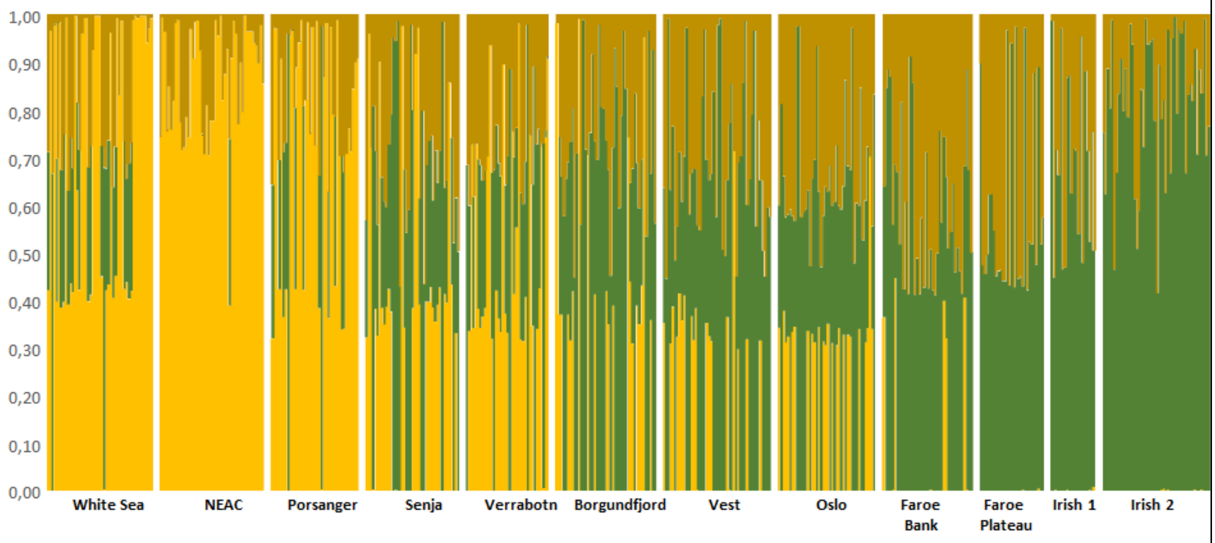

h) LG12, K =2

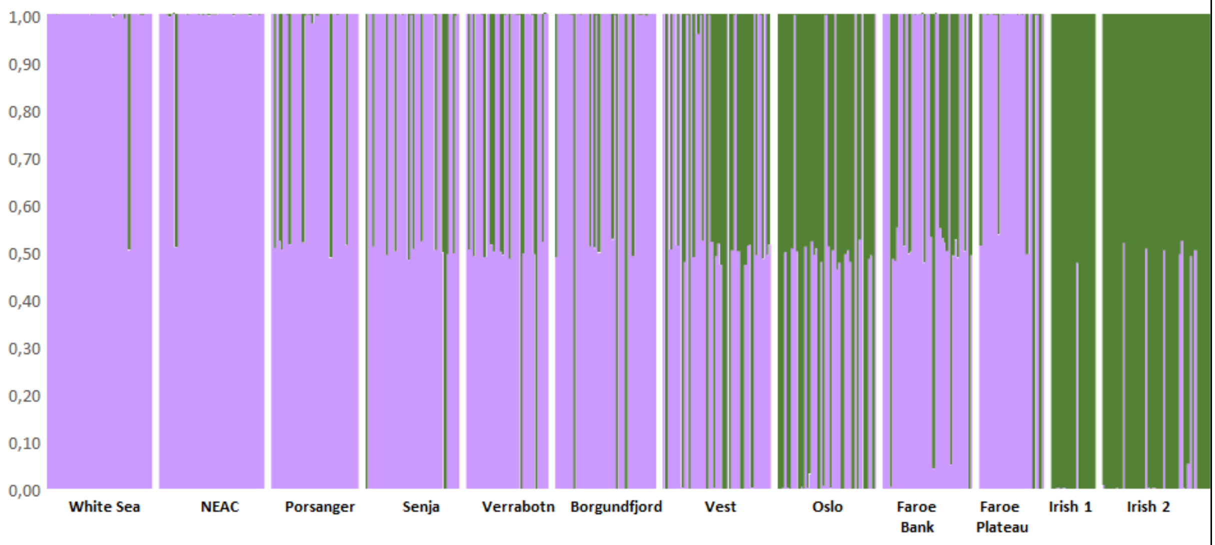

i) LG12, K=3

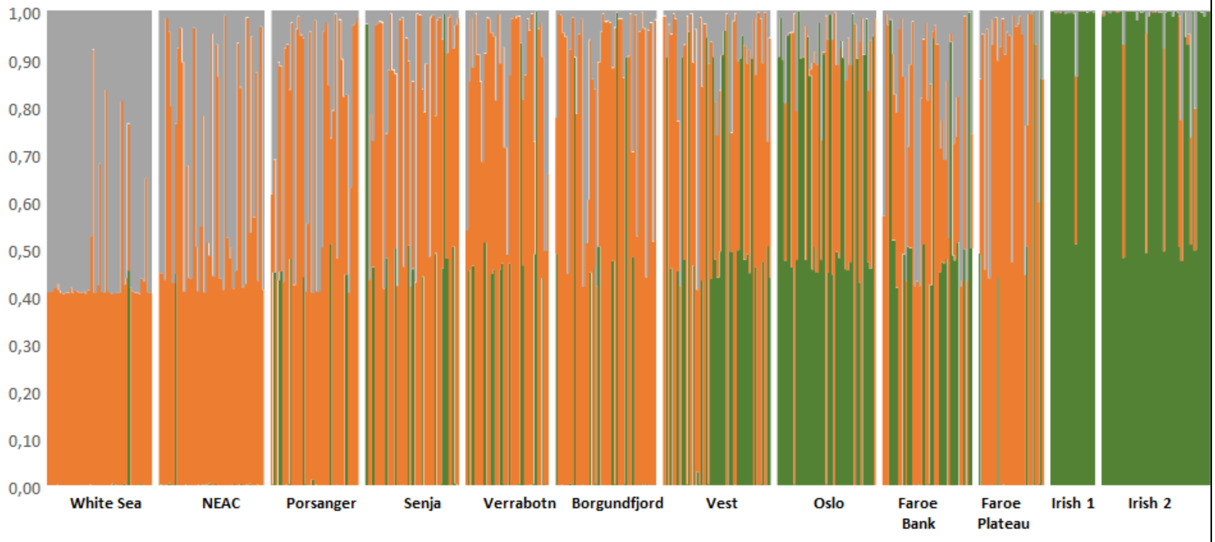

j) POOL SELECTED LOCI within LG1, 2, 7, 12; K=2

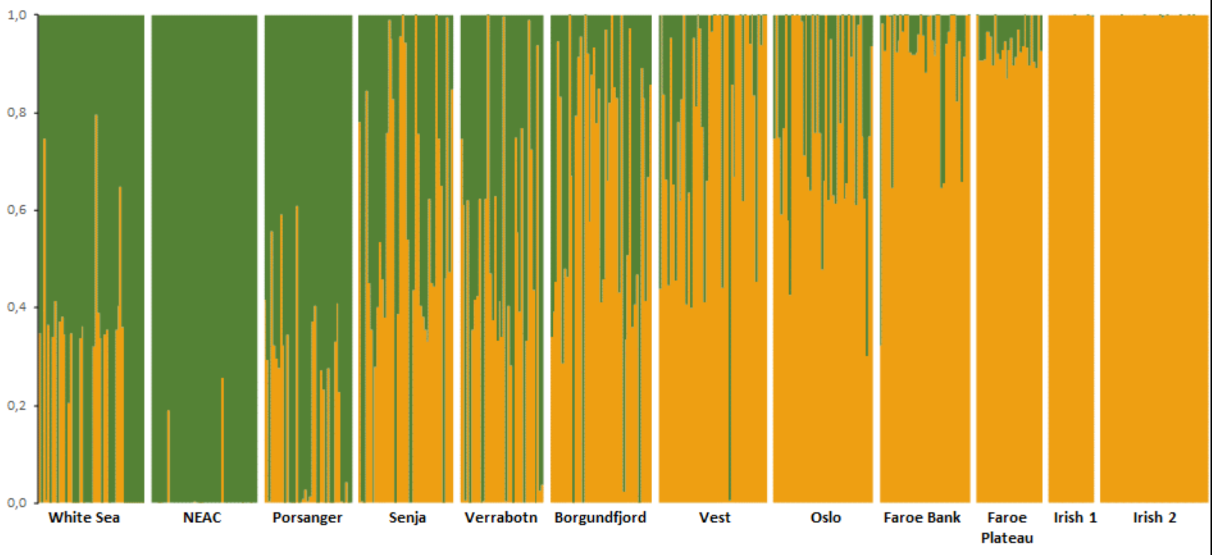

k) POOL SELECTED LOCI within LG1, 2, 7, 12; K=4

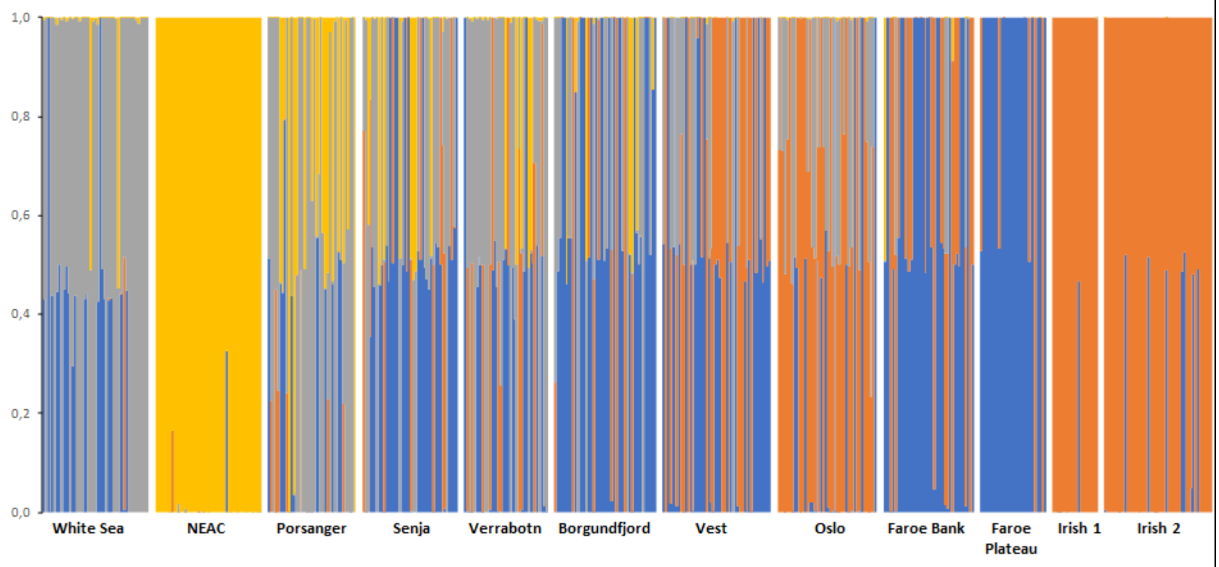

Figure S4. Bayesian clustering for the twelve populations genotyped at loci under positive selection. Inferred ancestry of individuals was calculated after averaging ten STRUCTURE runs with CLUMPP on the following datasets of selected loci: LG1 (barplots a-b). LG2 (barplots c-d-e). LG7 (barplot f-g) and LG12 (baplot h-i), pool of loci under selection within the four LG (barplots j-k). See Table S1 for Evanno test and StructureSelector results.

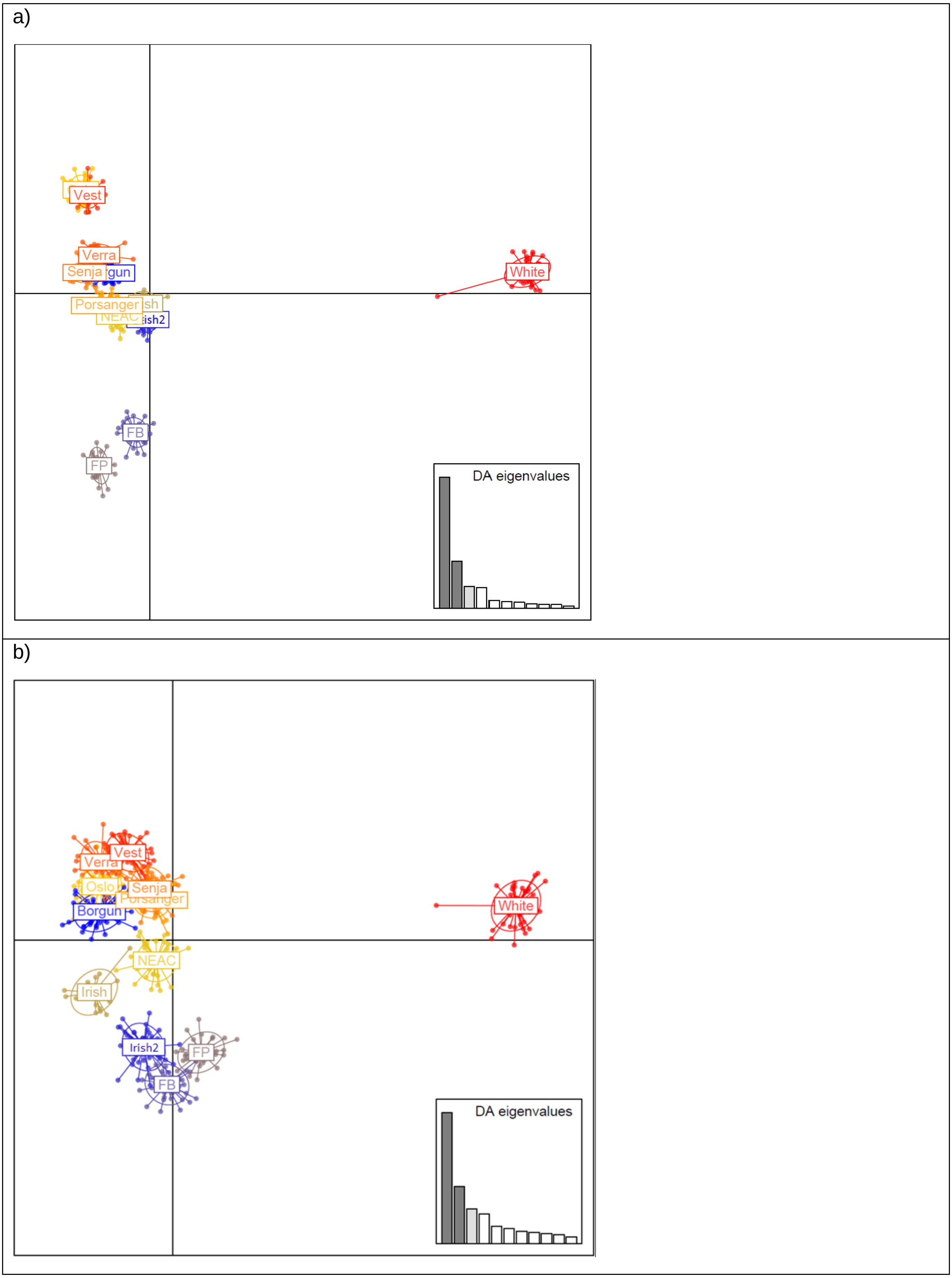

Figure S5. Discriminant Analyses of Principal Components (DAPC) of the samples from the twelve geographical regions based on neutral markers: a) 5854 SNPs (Neutral-A) and b) 1344 SNPs (Neutral-B).

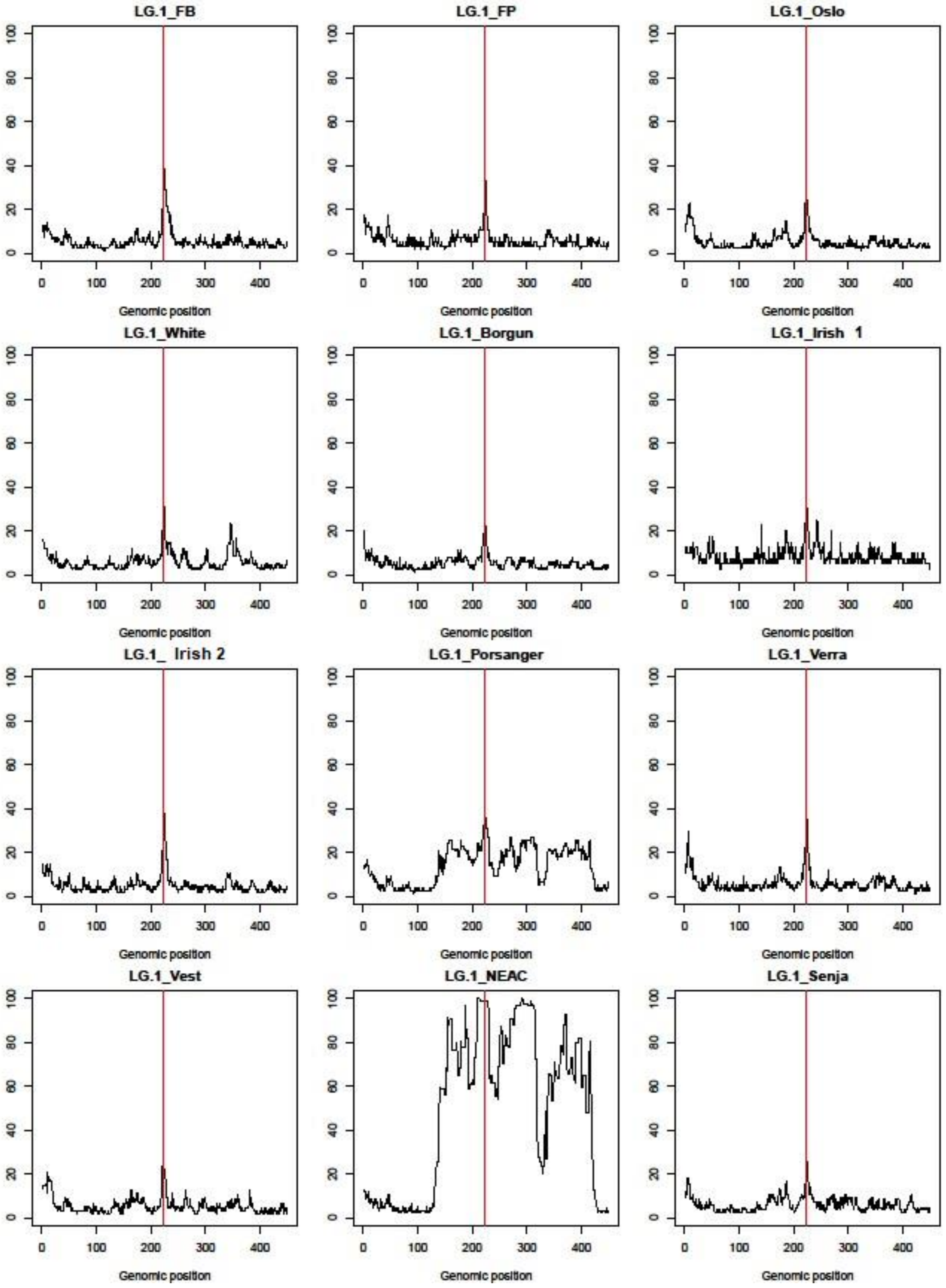

Figure S6. Haplotype reconstruction in 15-SNPs sliding windows along LG1. Each sub-plot corresponds to a different sample: The X-axis represents the position of each SNP on the LG, with the frequency of the most frequent haplotype at a given genomic region along the Y-axis. The red line depicts the position that is consistently conserved across sampling sites.

a)

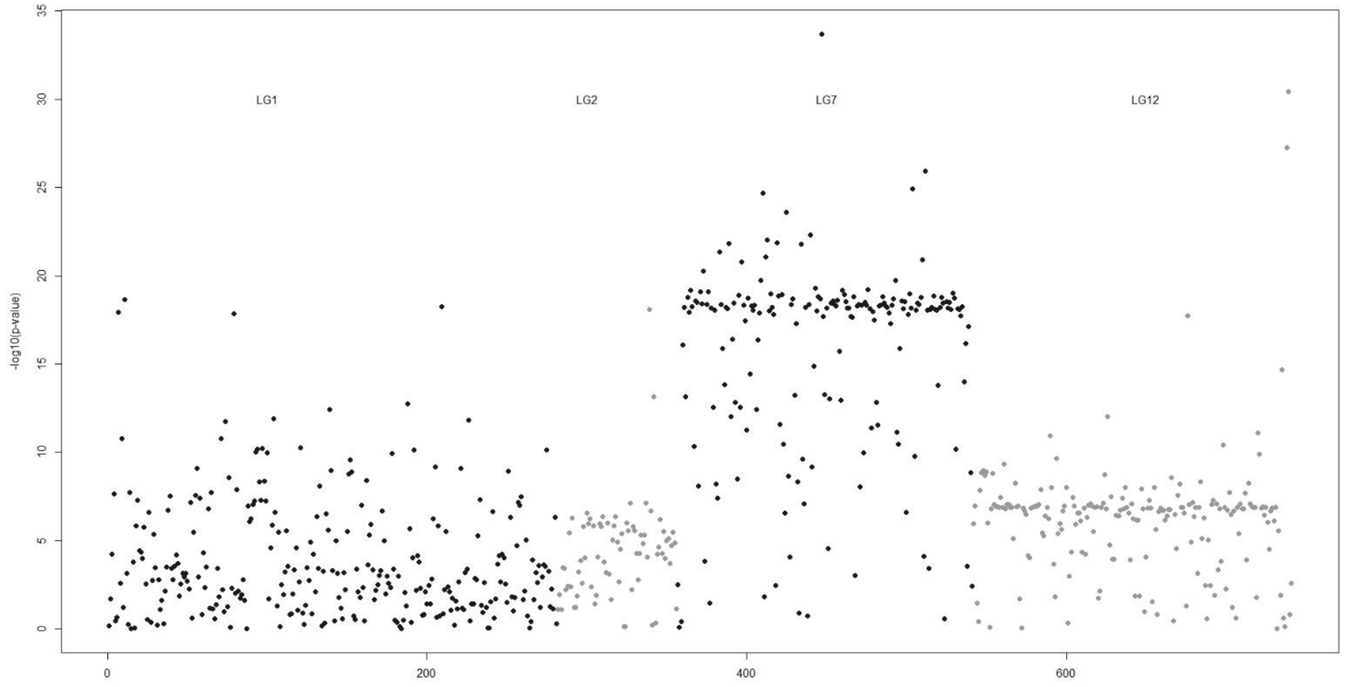

b)

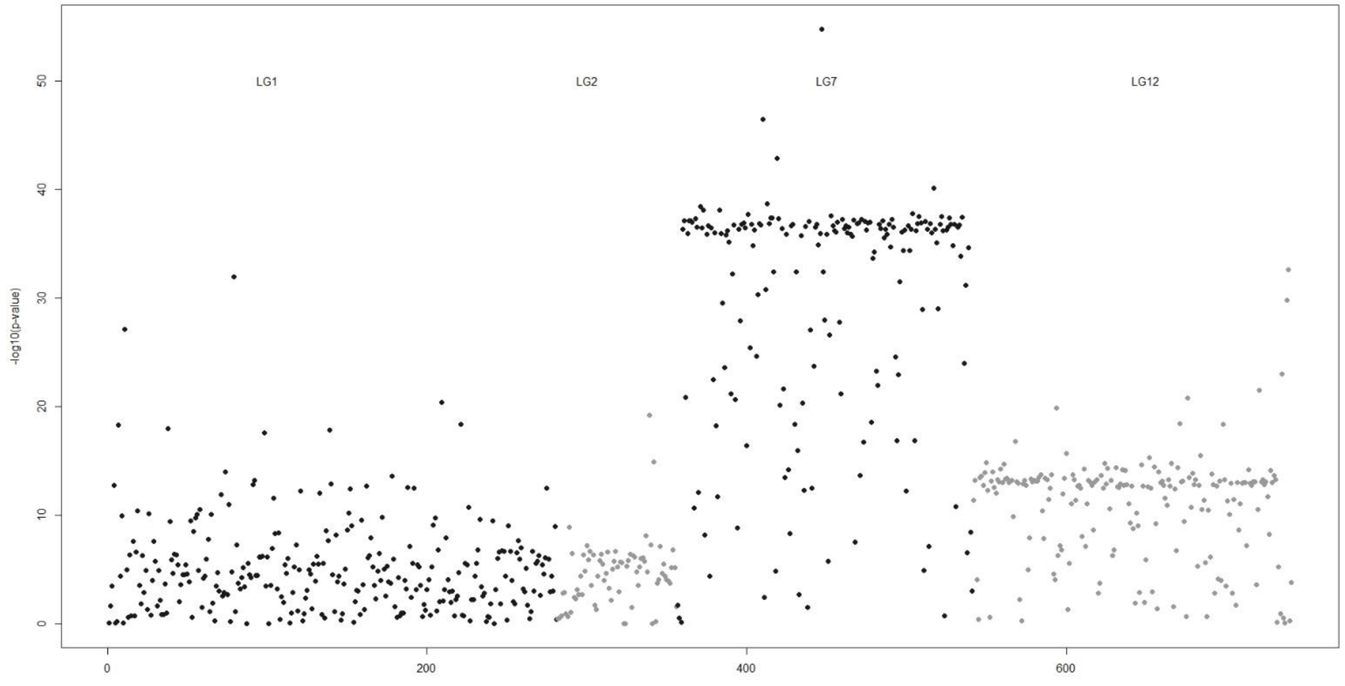

Figure S7. Associations between genetic variation of loci belonging to LG 1, 2, 7,12 and temperature measured at 50 m depth in the months of March (a) and July (b) assessed by LFMM.
